# Supplementary material for: Development of Dicationic Bisguanidine-Arylfuran Derivatives as Potent Agents against Gram-Negative Bacteria
Source: Antibiotics (Basel). 2022 Aug 17;11(8):1115. doi: 10.3390/antibiotics11081115 (PMC9404985; doi:10.3390/antibiotics11081115)
Supplement: Supplementary file 1 [file antibiotics-11-01115-s001.zip › antibiotics-1852437-supplementary.pdf]

Supporting Information

Development of Dicationic Bisguanidine-arylfuran Derivatives as Potent Agents Against Gram-negative Bacteria

Catarina Bourgard[1,2†], Diego Rodríguez-Hernández[1†], Anastasia Rudenko[1,2], Carolin Rutgersson[2,3], Martin Palm[1,2], D.G. Joakim Larsson[2,3], Anne Farewell[1,2], Morten Grøtli[1,*] and Per Sunnerhagen[1,2,*]

[1] Department of Chemistry and Molecular Biology, University of Gothenburg, S-405 30 Gothenburg, Sweden.
[2] Centre for Antibiotic Resistance Research (CARe), University of Gothenburg, S-405 30 Gothenburg, Sweden.
[3] Institute of Biomedicine, Department of Infectious Diseases, University of Gothenburg, S-413 46 Gothenburg, Sweden.
[†] These authors contributed equally to this work.
* Correspondence authors: grotli@chem.gu.se (M.G.); per.sunnerhagen@cmb.gu.se (P.S.)

Table of Contents

1. Scheme S1. Synthetic pathway to **1**, **3-9a** 3
2. Scheme S2. Synthetic pathway to **10a** 3
3. Scheme S3. Synthetic pathway to **11-14a** 3
4. Scheme S4. Synthetic pathways to **16-20a** 4
5. Table S1. Effective concentrations of each compound against the Gram-negative and Gram-positive set of laboratory strains 5
6. Table S2. Selectivity indexes of each compound against the Gram-negative and Gram-positive set of laboratory strains 6
7. Table S3. MIC values of established antibiotics for the clinical isolates of ESKAPE species and *E. coli* tested in this paper 7
8. Table S4. Genomic G/C content (%) of bacterial species 7
9. Figure S1. Cytotoxicity dose-response curves of all compounds against MCF-7 (A, B and C) and HepG2 (D, E and F) cell lines 8
10. Figure S2. Antibacterial activity dose-response curves of all compounds against Gram-negative *E. coli* (A, B and C), *P. putida* (D, E and F), *P. carotovorum* (G, H and I) and *P. caledonica* (J, K and L) 8
11. Figure S3. Antibacterial activity dose-response curves of all compounds against Gram-positive *B. subtilis* (A, B and C) 9
12. Figure S4. Measurements of the antibacterial activity of ampicillin, as a positive control antibiotic tested against Gram-negative *Escherichia coli* (A) and Gram-positive *Bacillus subtilis* (B) 10
13. Figure S5. Antibacterial activity dose-response curves of **1** against the 10 Gram-negative bacteria 10
14. Figure S6. Antibacterial activity dose-response curves of **3** against the 10 Gram-negative bacteria 11
15. Figure S7. Antibacterial activity dose-response curves of **4** against the 10 Gram-negative bacteria 11
16. Figure S8. Antibacterial activity dose-response curves of **6** against the 10 Gram-negative bacteria 12
17. Figure S9. Antibacterial activity dose-response curves of **8** against the 10 Gram-negative bacteria 12
18. Figure S10. Antibacterial activity dose-response curves of **10** against the 10 Gram-negative bacteria 13
19. Figure S11. Antibacterial activity dose-response curves of **16** against the 10 Gram-negative bacteria 13
20. Figure S12. Antibacterial activity dose-response curves of **17** against the 10 Gram-negative bacteria 14
21. Figure S13. Antibacterial activity dose-response curves of the known antibiotic cefotaxime (CTX) as a control against the 10 Gram-negative bacteria 14
22. Figure S14. High-resolution microbial phenomics profiling of the synthesized compounds **1** (A), **4** (B), **6** (C), **10** (D), and **16** (E), and the known antibiotic cefotaxime (F), against two *E. coli* antibiotic resistant libraries 15
23. Figures S15-S32. NMR spectra 16

SUPPLEMENTARY SCHEMES

Scheme S1. Synthetic pathway to **1**, **3**-**9**[a].

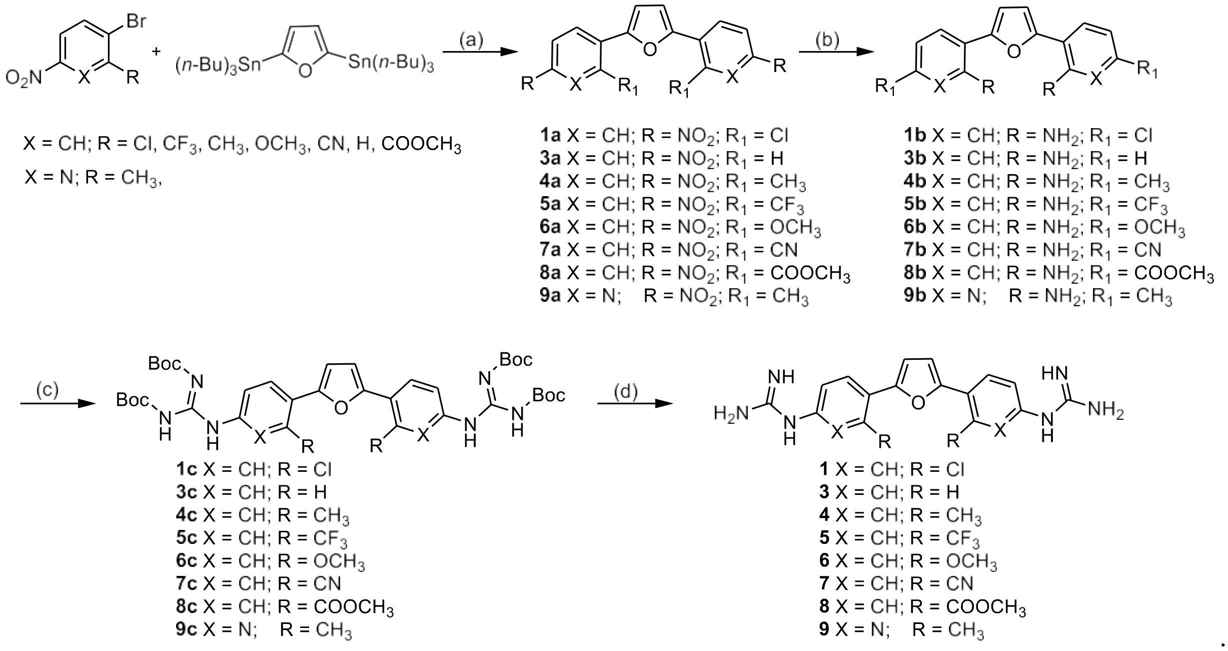

[a]*Reagents and conditions*: (a) $Pd(PPh_3)_4$, DMF, 100 °C, 14 h, 46-80%; (b) Fe, NH_4Cl, EtOH, THF, 60 °C, 4 h, 75-95%; (c) 1,3-bis(*tert*-butoxicarbonyl)-2-methyl-2-thiopseudourea, $HgCl_2$, TEA, DMF, rt, 16 h, 93-95%; (d) 4 M HCl in Dioxane, rt, 12 h, 86-90%.

Scheme S2. Synthetic pathway to **10**[a].

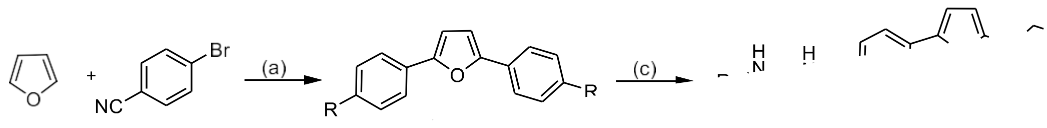

[a]*Reagents and conditions*: (a) $Pd(OAc)_2$, KOAc, DMA, 150 °C, 20 h, 29%; (b) LAH, THF, rt, 24 h, 60%; (c) 1,3-bis(*tert*-butoxi-carbonyl)-2-methyl-2-thiopseudourea, $HgCl_2$, TEA, DMF, rt, 16 h, 76%; (d) 4 M HCl in Dioxane, rt, 12 h, 79%.

Scheme S3. Synthetic pathway to **11**-**14**[a].

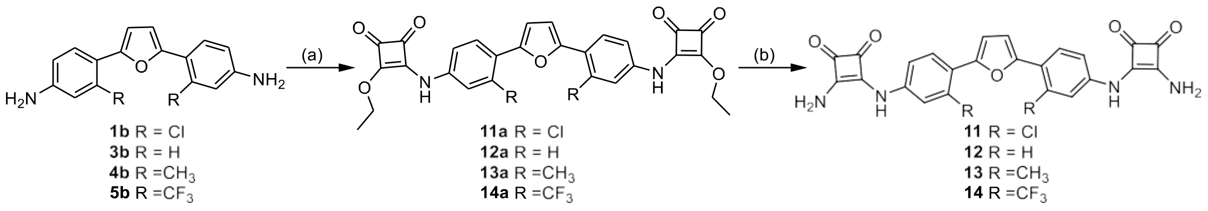

[a]*Reagents and conditions:* (a) 3,4-diethoxycyclobut-3-ene-1,2-dione, $(Zn(OTf)_2$, EtOH, rt, 4 h, 36-75%; (b) NH_3 in MeOH 7M, MeOH, rt, 4 h, 52-77%.

Scheme S4. Synthetic pathways to **16**-**20**[a].

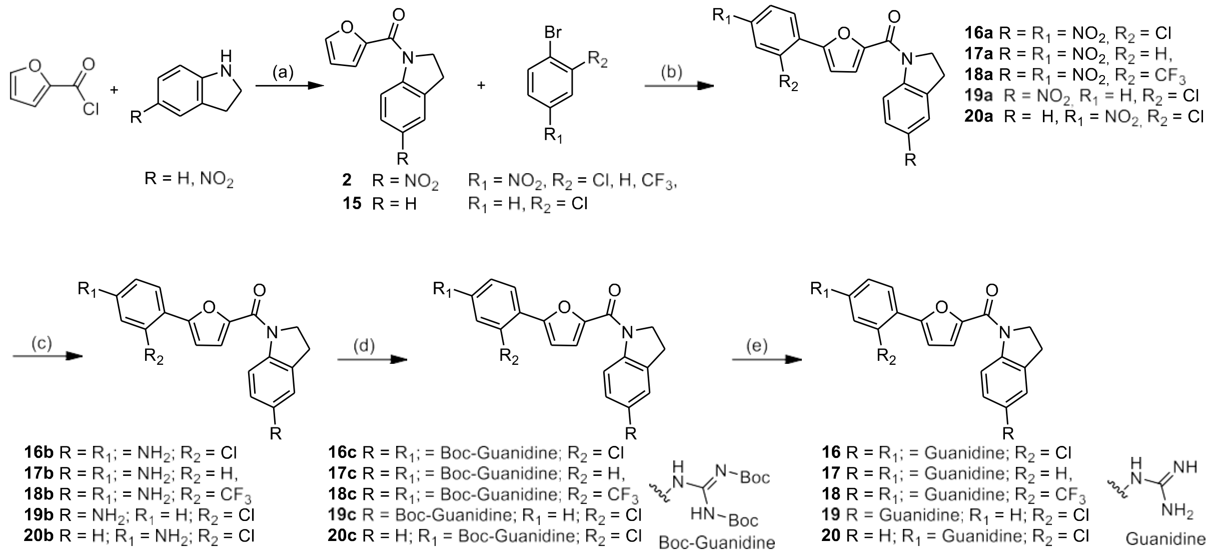

[a]*Reagents and conditions*: (a) TEA, DCM, rt, 4 h, 50%; (b) Pd(OAc)$_2$, KOAc, DMA, 150 °C, 20 h, 43-50%; (c) Fe, NH_4Cl, EtOH, THF, 60 °C, 4 h, 75-84%; (d) 1,3-bis(*tert*-butoxicarbonyl)-2-methyl-2-thiopseudourea, $HgCl_2$, TEA, DMF, rt, 16 h, 21-45%; (e) 4 M HCl in Dioxane, rt, 12 h, 35-86%.

SUPPLEMENTARY TABLES

Table S1. Effective concentrations of each compound against the Gram-negative and Gram-positive set of laboratory strains.

Structure	#	Bsu		Eco		Ppu		Pcar		Pcal	
		EC50	EC90	EC50	EC90	EC50	EC90	EC50	EC90	EC50	EC90
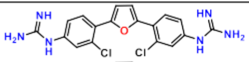	**1**	25.4	136.1	64.8	148.2	257.8	517.1	67.1	114.9	20.3	135.9
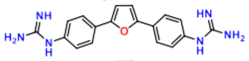	**3**	11.2	22.5	80.4	173.8	8.3	14.3	34.6	79.0	4.5	18.9
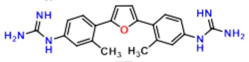	**4**	6.2	8.5	48.9	80.8	14.5	59.6	28.6	37.3	8.2	51.9
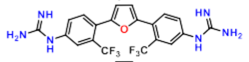	**5**	NE	NE	29.4	59.1	290.3	319.1	184.9	233.2	NE	NE
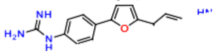	**6**	NE	NE	35.7	76.3	14.3	19.7	28.4	45.0	25.6	35.6
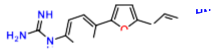	**7**	146.2	562.5	59.33	349.7	24.5	51.5	212.3	268.1	NE	NE
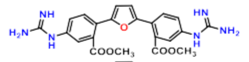	**8**	22.5	43.6	95.4	128.6	68.4	92.1	140.0	157.5	NE	NE
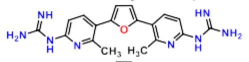	**9**	154.2	245.8	547.3	>900	NE	NE	NE	NE	84.8	212.6
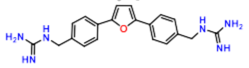	**10**	NE	NE	73.2	108.4	25.6	36.7	43.6	61.2	2.9	5.0
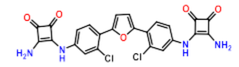	**11**	NE	NE	NE	NE	NE	NE	NE	NE	19.0	60.1
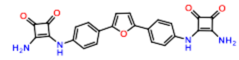	**12**	NE	NE	NE	NE	NE	NE	NE	NE	NE	NE
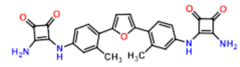	**13**	NE	NE	NE	NE	NE	NE	NE	NE	NE	NE
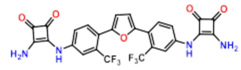	**14**	NE	NE	NE	NE	NE	NE	NE	NE	90.4	304.9
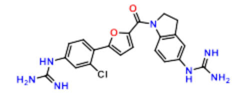	**16**	NE	NE	13.1	19.0	94.2	147.0	70.9	80.7	51.4	175.6
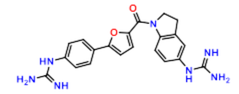	**17**	NE	NE	1.7	4.3	73.9	98.0	129.9	142.4	72.5	>300
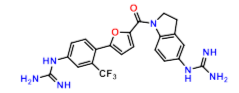	**18**	9.6	13.6	42.3	58.3	10.1	22.2	94.6	120.2	12.6	176.0
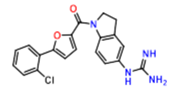	**19**	14.5	25.9	0.5	2.5	75.0	84.9	87.1	130.1	96.3	287.0
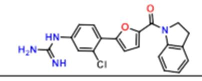	**20**	24.5	38.4	61.7	105.8	NE	NE	158.9	186.6	129.4	>300

Half maximal effective concentration (EC50) and 90% maximal effective concentration (EC90) are expressed in μM for each compound. Values >900 and >300 μM represent the maximum compound concentration tested in the assays, without observing a 50% or 90% inhibition. NE, No effective concentration observed. Tested organisms were *B. subtilis* (Bsu); *E. coli* (Eco); *P. putida* (Ppu); *P. carotovorum* (Pcar); *P. caledonica* (Pcal).

Table S2. Selectivity indexes of each compound against the Gram-negative and Gram-positive set of laboratory strains.

Code	B. subtilis	E. coli	P. putida	P. carotovorum	P. caledonica
**1**	1.5	0.6	0.2	0.6	1.9
**3**	11.1	1.5	15.0	3.6	27.4
**4**	12.2	1.5	5.2	2.6	9.2
**5**	NE	1.6	0.2	0.2	NE
**6**	NE	4.0	10.1	5.1	5.6
**7**	0.4	1.1	2.5	0.3	NE
**8**	10.8	2.5	3.6	1.7	NE
**9**	3.6	1.0	NE	NE	6.5
**10**	NE	1.3	3.6	2.1	31.8
**11**	NE	NE	NE	NE	18.2
**12**	NE	NE	NE	NE	NE
**13**	NE	NE	NE	NE	NE
**14**	NE	NE	NE	NE	2.0
**16**	NE	16.0	2.2	2.9	4.1
**17**	NE	114.3	2.6	1.5	2.7
**18**	32.9	7.5	31.3	3.3	25.1
**19**	1.8	51.2	0.3	0.3	0.3
**20**	1.4	0.5	NE	0.2	0.3

Average selectivity indexes are calculated as the mean between the ratios of cytotoxicity EC_{50} (μM) verified for each human cell line (MCF-7 and HepG2) over each bacterial strains' EC_{50} (μM) values, SI = ((MCF-7 EC_{50}/bacterial strain EC_{50})+(HepG2 EC_{50}/bacterial strain EC_{50}))/2. The highest the value, the more selective is the compound against the different bacterial strain. *Cases where the maximum compound concentration assayed against the bacterial strain did not allow the observation of a half maximal effect concentration. NE, No Effective concentration observed.

Table S3. MIC values of established antibiotics for the ESKAPE strains tested in this paper as established by micro-broth dilution tests.

		MIC (μM)			
	**CCUG #**	**Amoxicillin**	**Ciprofloxacin**	**Cefotaxime**	**Meropenem**
**Escherichia coli**	17620	11-44	0.025	0.25-0.50	0.16
	67180	350	190	280	10-20
**CB (S ≤ , R ≥)**		22 , 22	0.75 , 1.5	2 , 4	5.2 , 20
**Klebsiella pneumoniae**	225T	350	0.18	0.25	0.16-0.32
	58547	350	190	560	>80
**CB (S ≤ , R ≥)**		22 , 22	0.75 , 1.5	2 , 4	5.2 , 20
**Pseudomonas aeruginosa**	17619	350	1.5-3	36	2.6-5.2
	59347	350	100	>560	335
**CB (S ≤ , R ≥)**		-	0.003 , 1.5	-	5.2 , 20
**Acinetobacter baumannii**	57250	175	200	36	40
	57035	175	200	>560	335
**CB (S ≤ , R ≥)**		-	0.003 , 3	-	5.2 , 20
**Enterobacter cloacae resp. hormaechei**	6323T	350	0.025-0.050	9-36	0.6-1.2
	58962	350	0.1	280	0.3
**CB (S ≤ , R ≥)**		22 , 22	0.75 , 1.5	2 , 4	5.2 , 20

Minimal Inhibitory Concentration (MIC) are expressed in μM for each antibiotic.
When applicable, clinical breakpoint concentrations (CB) as defined by EUCAST 2022-01-01 (https://www.eucast.org/clinical_breakpoints/) are indicated in μM.

Table S4. Genomic G/C content of bacterial species (%).

	C/G (%)	NCBI Reference Sequence
**Escherichia coli**	50.8	NC_000913.3
**Klebsiella pneumoniae**	57.1	NC_016845.1
**Acinetobacter baumannii**	39	NZ_CP043953.1
**Pseudomonas aeruginosa**	66.2	NC_002516.2
**Enterobacter cloacae**	55	NZ_CP009756.1
**Enterobacter hormaechei**	55	GCF_000694955.1
**Bacillus subtilis**	43.5	NC_000964.3
**Pectobacterium carotovorum**	51.9	NZ_CP051652.1
**Paraburkholderia caledonica**	61.9	GCF_000383275.1
**Pseudomonas putida**	61.9	NC_021505.1

SUPPLEMENTARY FIGURES

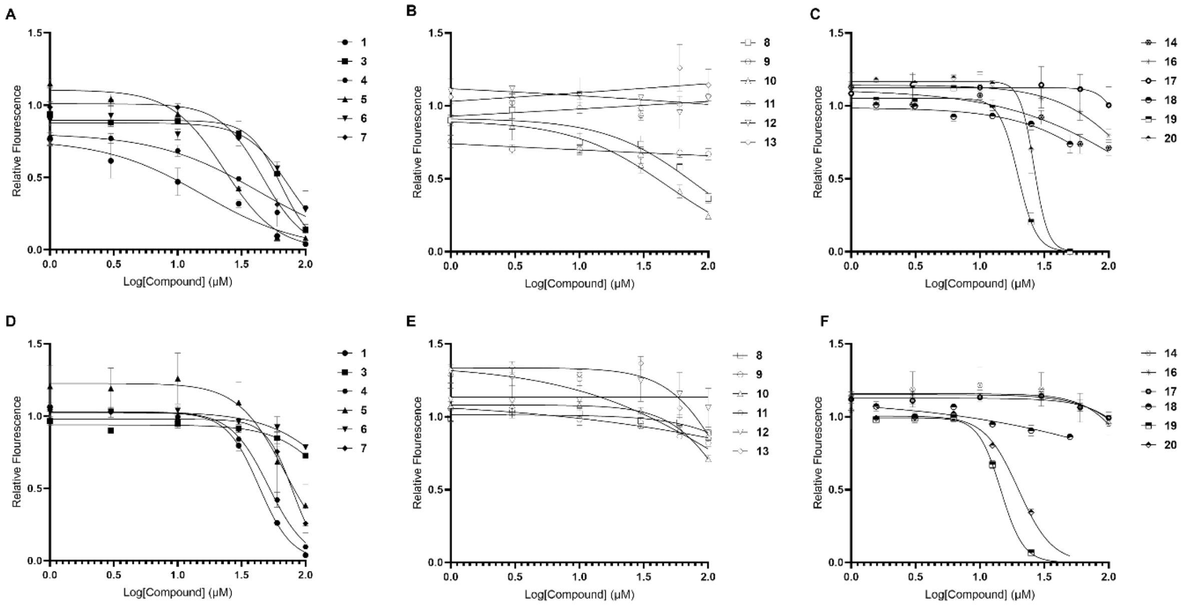

Figure S1. Cytotoxicity dose-response curves of all compounds against MCF-7 (**A**, **B** and **C**) and HepG2 (**D**, **E** and **F**) cell lines. The mean half maximal effective concentrations (EC_{50} in μM) for each compound against the correspondent cell lines were calculated using non-linear regression dose-response inhibition following a log(agonist) vs. response, shown in Table 1. Error bars represent the SEM.

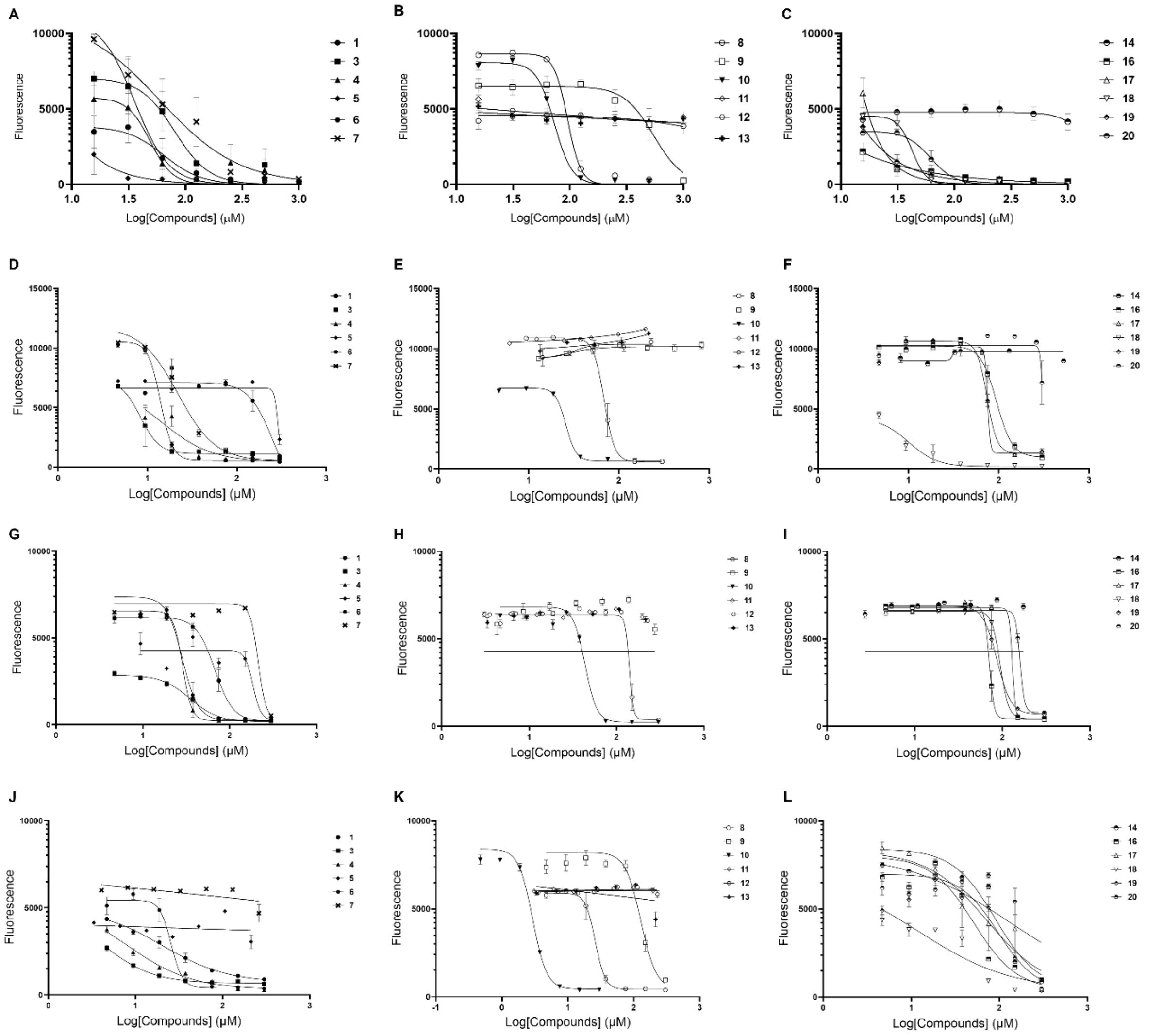

Figure S2. Antibacterial activity dose-response curves of all compounds against Gram-negative *E. coli* (**A**, **B** and **C**), *P. putida* (**D**, **E** and **F**), *P. carotovorum* (**G**, **H** and **I**) and *P. caledonica* (**J**, **K** and **L**). EC_{50} and EC_{90} values (in μM) determined for each compound can be found in Table S1.

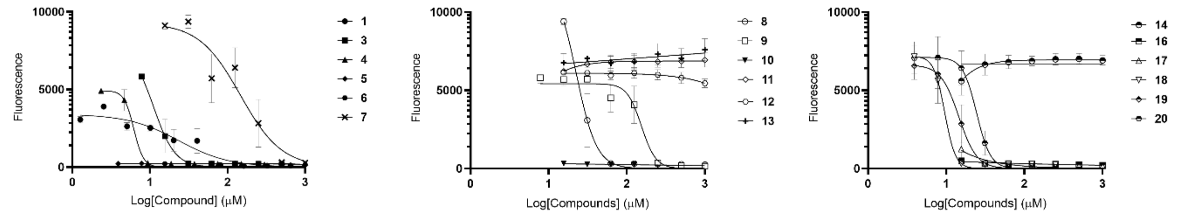

Figure S3. Antibacterial activity dose-response curves of all compounds against Gram-positive *B. subtilis* (**A, B** and **C**). EC_{50} and EC_{90} values (in μM) determined for each compound can be found in Table S1.

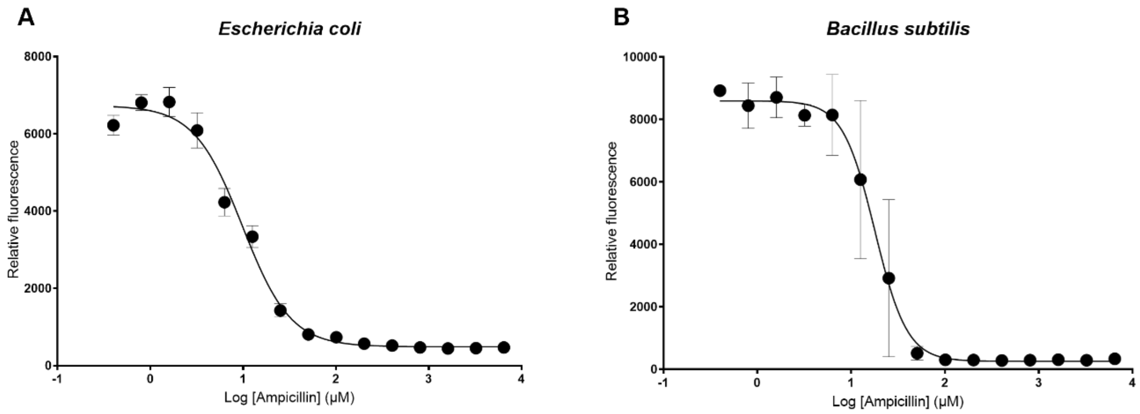

Figure S4. Measurements of the antibacterial activity of ampicillin, as a positive control antibiotic tested against Gram-negative *Escherichia coli* (**A**) and Gram-positive *Bacillus subtilis* (**B**). Ampicillin showed an EC_{50} = 11.5 μM (SD = 0.72; SE range 11.1 to 11.9 μM) and an EC_{90} = 89.2 μM (SD = 14.8; SE range 81.5 to 98.6 μM) against *E. coli* and an EC_{50} = 17.2 μM (SD = 8.9; SE range 13.5 to 23.8 μM) and an EC_{90} = 160.4 μM (SD = 42.8; SE range 97.1 to 146.5 μM) against *B. subtilis*, as previously reported (European Committee on Antimicrobial Susceptibility Testing. Data from the EUCAST MIC distribution website, last accessed November 2020". http://www.eucast.org).

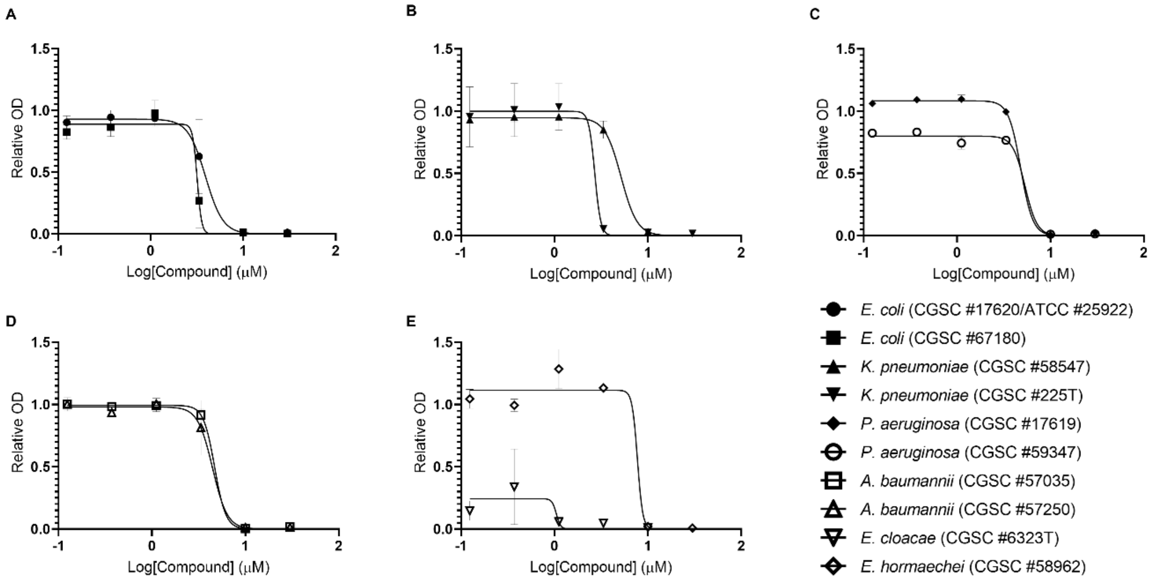

Figure S5. Antibacterial activity dose-response curves of **1** against the 10 Gram-negative bacteria *E. coli* CCUG #67180 and CCUG #17620/ATCC #25922 (control strain) (**A**), *K. pneumoniae* CCUG #58547 and CCUG #225T (**B**), *P. aeruginosa* CCUG #17619 and CCUG #59347 (**C**), *A. baumannii* CCUG #57035 and CCUG #57250 (**D**), *E. cloacae* CCUG #6323T and *E. hormaechei* CCUG #58962 (**E**). EC_{50} and EC_{90} values (in μM) can be found in **Table 2**.

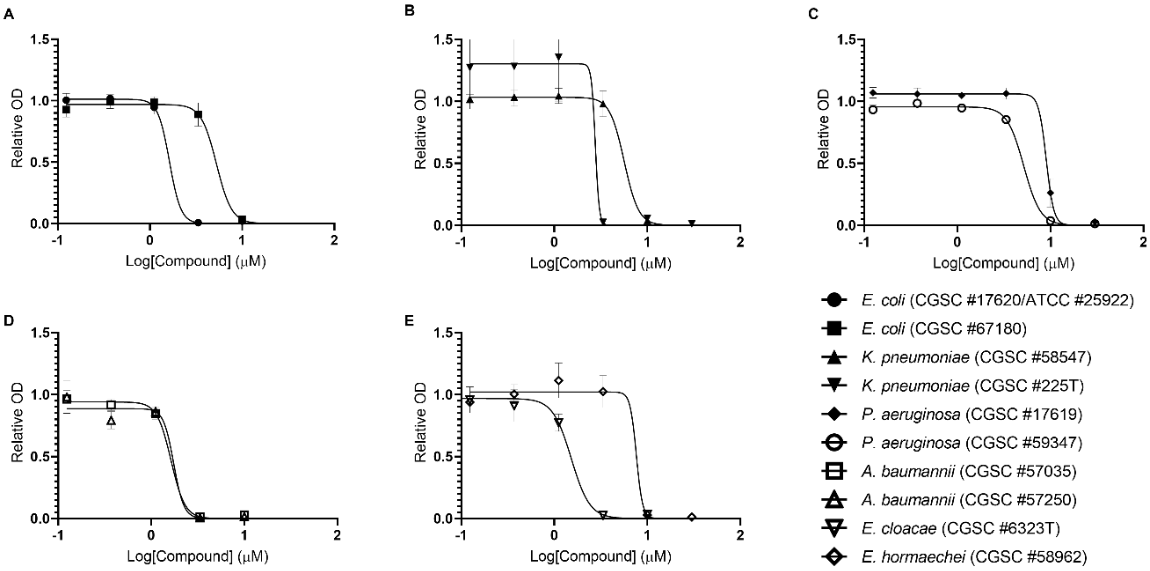

Figure S6. Antibacterial activity dose-response curves of **3** against the 10 Gram-negative bacteria *E. coli* CCUG #67180 and CCUG #17620/ATCC #25922 (control strain) (**A**), *K. pneumoniae* CCUG #58547 and CCUG #225T (**B**), *P. aeruginosa* CCUG #17619 and CCUG #59347 (**C**), *A. baumannii* CCUG #57035 and CCUG #57250 (**D**), *E. cloacae* CCUG #6323T and *E. hormaechei* CCUG #58962 (**E**). EC_{50} and EC_{90} values (in μM) can be found in Table 2.

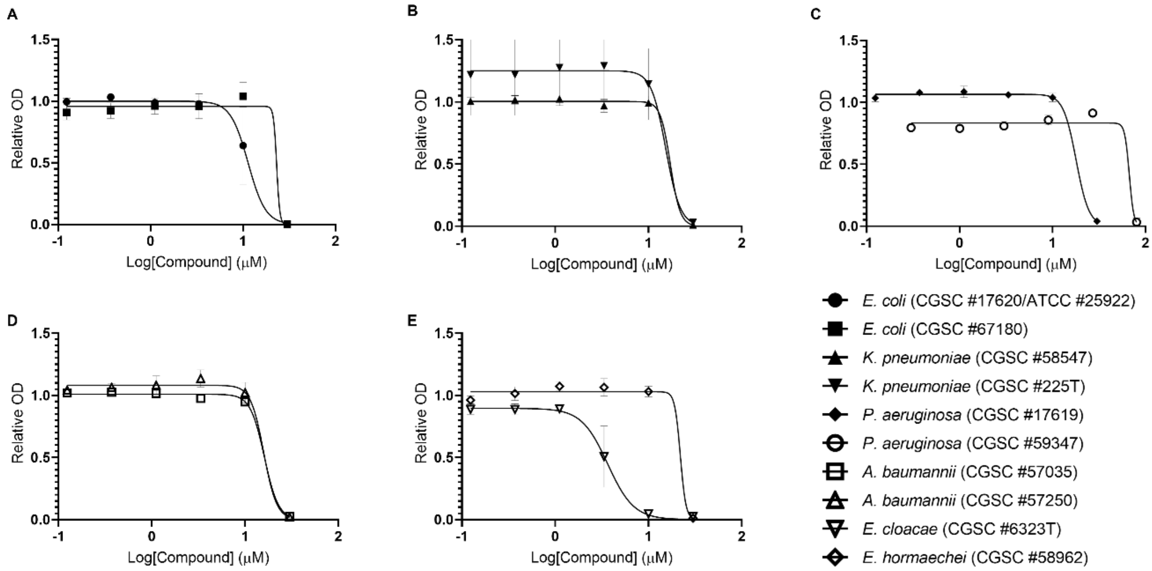

Figure S7. Antibacterial activity dose-response curves of **4** against the 10 Gram-negative bacteria *E. coli* CCUG #67180 and CCUG #17620/ATCC #25922 (control strain) (**A**), *K. pneumoniae* CCUG #58547 and CCUG #225T (**B**), *P. aeruginosa* CCUG #17619 and CCUG #59347 (**C**), *A. baumannii* CCUG #57035 and CCUG #57250 (**D**), *E. cloacae* CCUG #6323T and *E. hormaechei* CCUG #58962 (**E**). EC_{50} and EC_{90} values (in μM) can be found in Table 2.

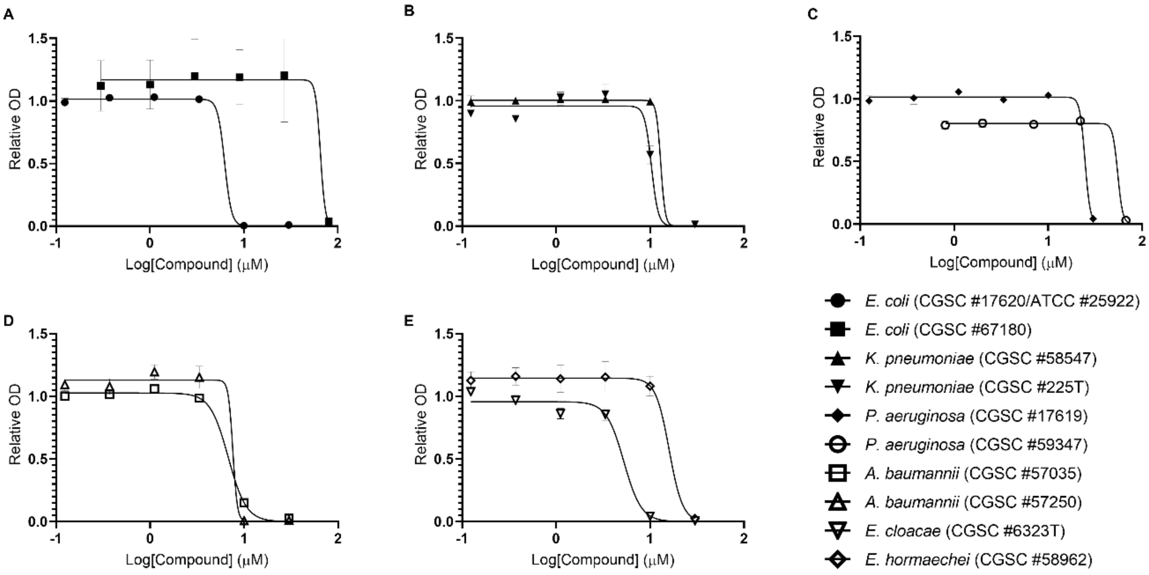

Figure S8. Antibacterial activity dose-response curves of **6** against the 10 Gram-negative bacteria *E. coli* CCUG #67180 and CCUG #17620/ATCC #25922 (control strain) (**A**), *K. pneumoniae* CCUG #58547 and CCUG #225T (**B**), *P. aeruginosa* CCUG #17619 and CCUG #59347 (**C**), *A. baumannii* CCUG #57035 and CCUG #57250 (**D**), *E. cloacae* CCUG #6323T and *E. hormaechei* CCUG #58962 (**E**). EC_{50} and EC_{90} values (in μM) can be found in Table 2.

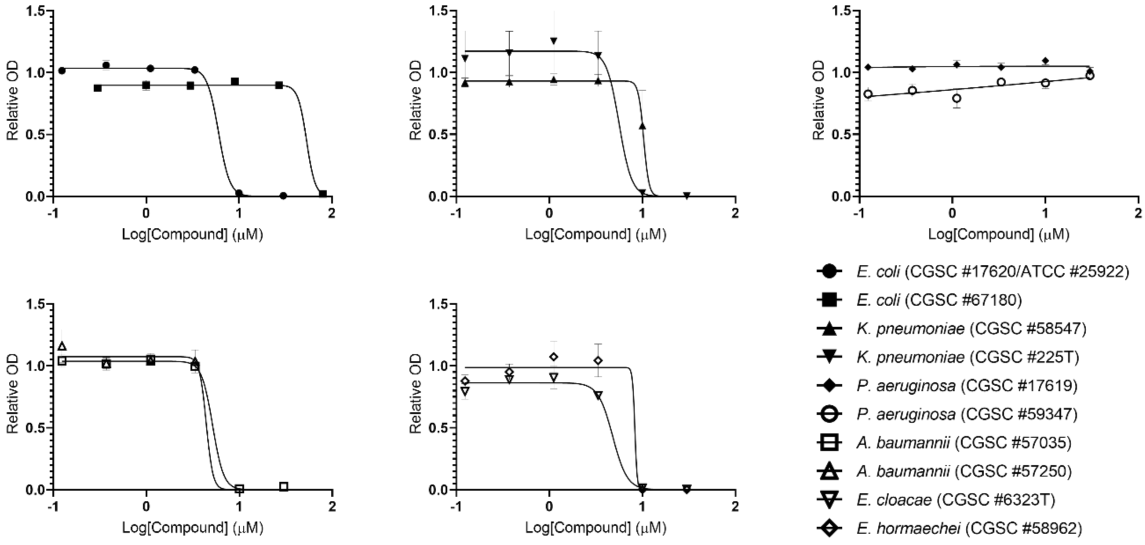

Figure S9. Antibacterial activity dose-response curves of **8** against the 10 Gram-negative bacteria *E. coli* CCUG #67180 and CCUG #17620/ATCC #25922 (control strain) (**A**), *K. pneumoniae* CCUG #58547 and CCUG #225T (**B**), *P. aeruginosa* CCUG #17619 and CCUG #59347 (**C**), *A. baumannii* CCUG #57035 and CCUG #57250 (**D**), *E. cloacae* CCUG #6323T and *E. hormaechei* CCUG #58962 (**E**). EC_{50} and EC_{90} values (in μM) can be found in Table 2.

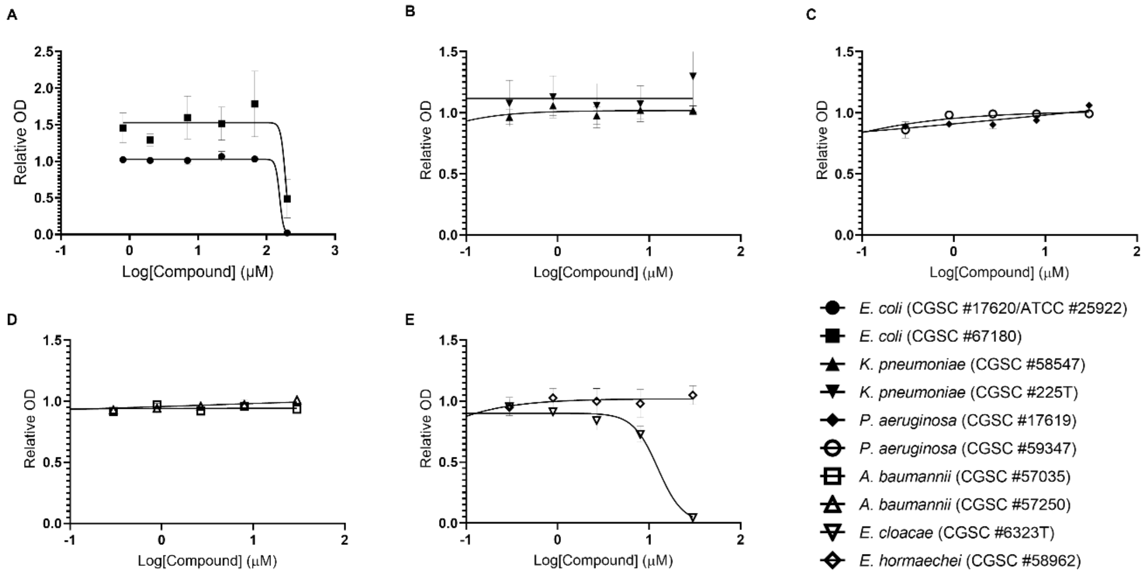

Figure S10. Antibacterial activity dose-response curves of **10** against the 10 Gram-negative bacteria *E. coli* CCUG #67180 and CCUG #17620/ATCC #25922 (control strain) (**A**), *K. pneumoniae* CCUG #58547 and CCUG #225T (**B**), *P. aeruginosa* CCUG #17619 and CCUG #59347 (**C**), *A. baumannii* CCUG #57035 and CCUG #57250 (**D**), *E. cloacae* CCUG #6323T and *E. hormaechei* CCUG #58962 (**E**). EC_{50} and EC_{90} values (in μM) can be found in Table 2.

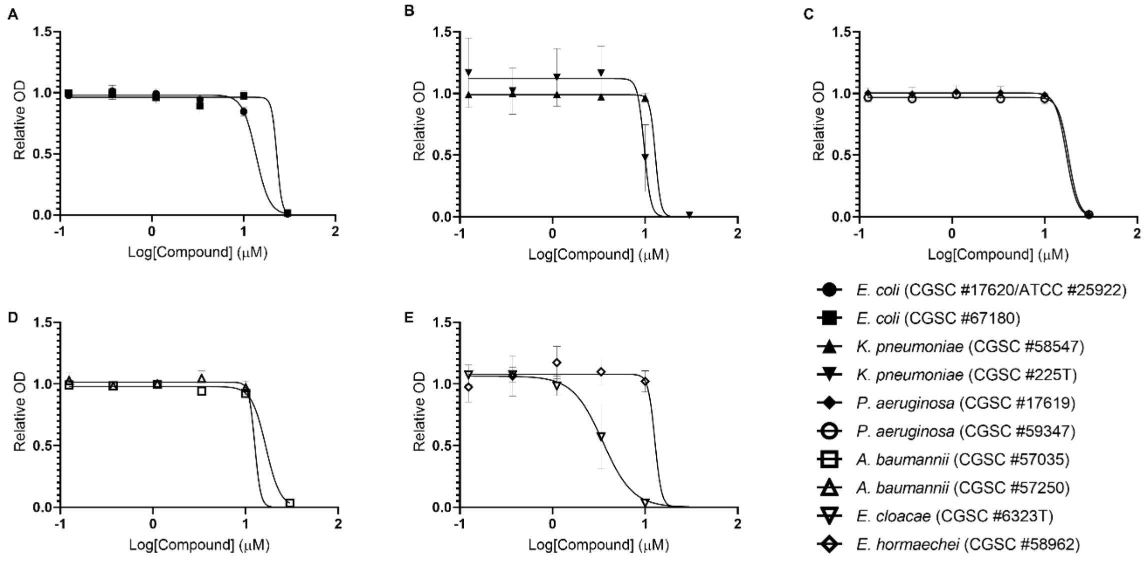

Figure S11. Antibacterial activity dose-response curves of **16** against the 10 Gram-negative bacteria *E. coli* CCUG #67180 and CCUG #17620/ATCC #25922 (control strain) (**A**), *K. pneumoniae* CCUG #58547 and CCUG #225T (**B**), *P. aeruginosa* CCUG #17619 and CCUG #59347 (**C**), *A. baumannii* CCUG #57035 and CCUG #57250 (**D**), *E. cloacae* CCUG #6323T and *E. hormaechei* CCUG #58962 (**E**). EC_{50} and EC_{90} values (in μM) can be found in Table 2.

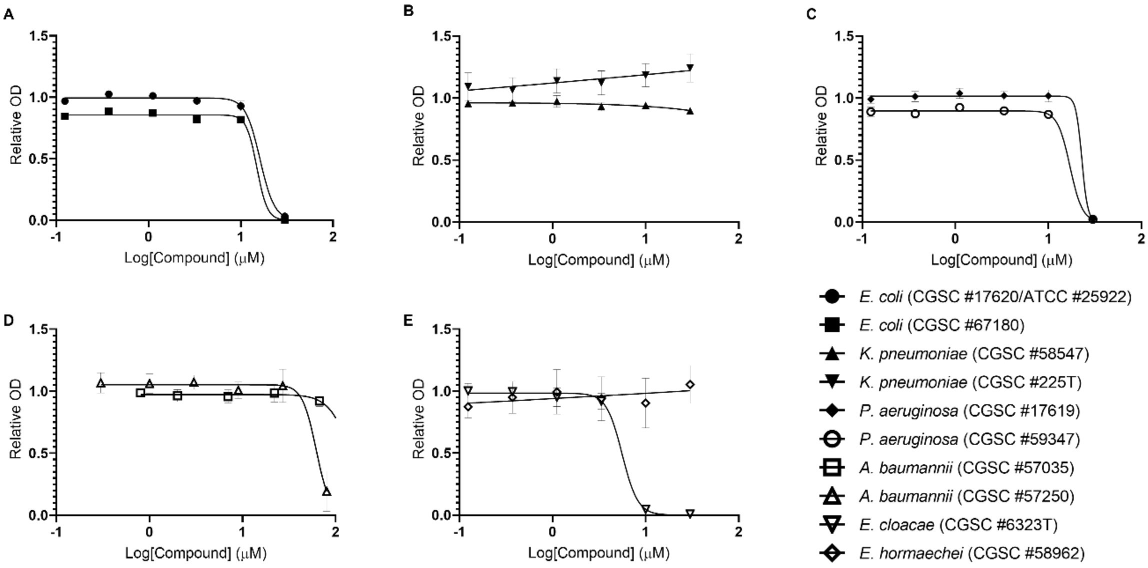

Figure S12. Antibacterial activity dose-response curves of **17** against the 10 Gram-negative bacteria *E. coli* CCUG #67180 and CCUG #17620/ATCC #25922 (control strain) (**A**), *K. pneumoniae* CCUG #58547 and CCUG #225T (**B**), *P. aeruginosa* CCUG #17619 and CCUG #59347 (**C**), *A. baumannii* CCUG #57035 and CCUG #57250 (**D**), *E. cloacae* CCUG #6323T and *E. hormaechei* CCUG #58962 (**E**). EC_{50} and EC_{90} values (in μM) can be found in Table 2.

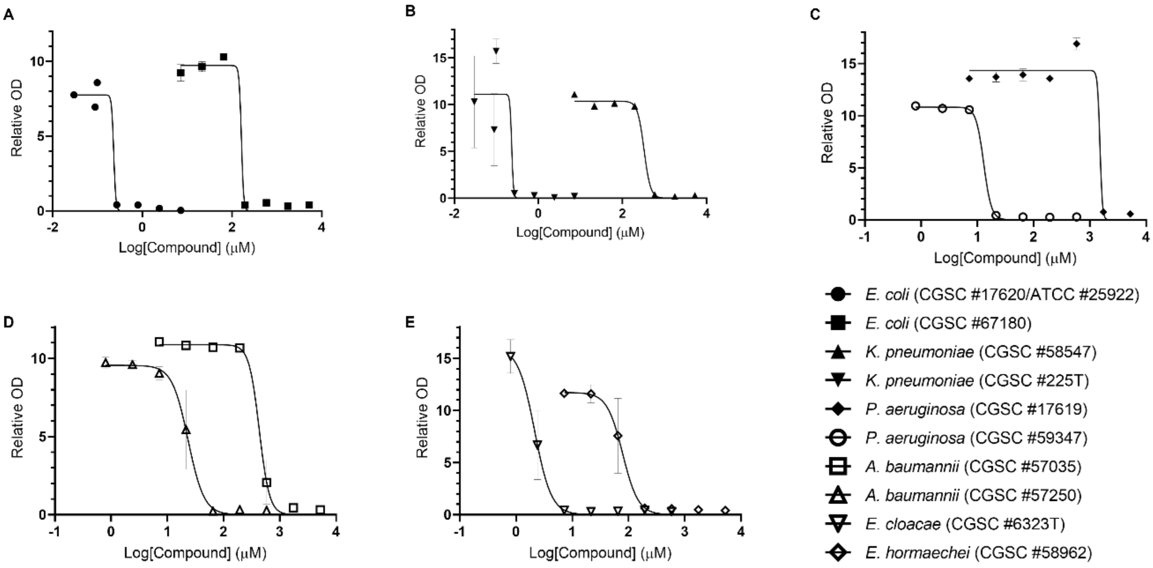

Figure S13. Antibacterial activity dose-response curves of the known antibiotic Cefotaxime (CTX) as a control against the 10 Gram-negative bacteria *E. coli* CCUG #67180 and CCUG #17620/ATCC #25922 (control strain) (**A**), *K. pneumoniae* CCUG #58547 and CCUG #225T (**B**), *P. aeruginosa* CCUG #17619 and CCUG #59347 (**C**), *A. baumannii* CCUG #57035 and CCUG #57250 (**D**), *E. cloacae* CCUG #6323T and *E. hormaechei* CCUG #58962 (**E**). EC_{50}, EC_{90} and SI values (in μM) can be found in Table 2.

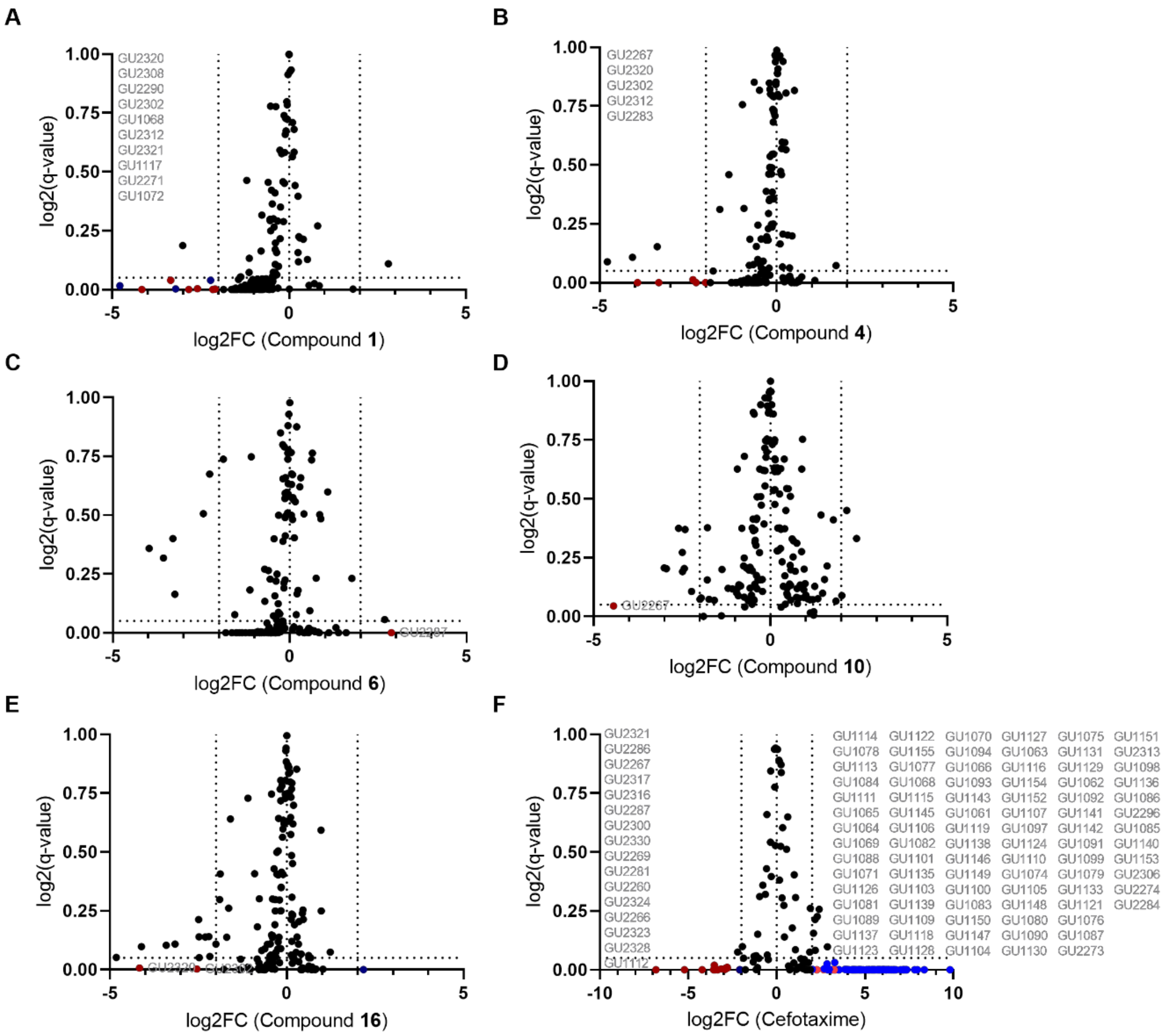

Figure S14. High-resolution microbial phenomics profiling of the synthesized compounds **1** (**A**), **4** (**B**), **6** (**C**), **10** (**D**) and **16** (**E**), and the known antibiotic cefotaxime (**F**) against two *E. coli* antibiotic resistant libraries. Volcano plots show a total of 168 strains screened from the ECOR (n=72) and ESBL (n=96) libraries. The strains presenting a differential yield growth relative to the reference strain *E. coli* CCUG #17620/ATCC #25922 are shown, where statistically significant strains more sensitive fall within a log2FC ≥ -2.0 and -log2(q-value ≤ 0.05) (left quadrant in light red for ECOR and light blue for ESBL libraries) and resistant within a log2FC ≥ 2.0 and -log2(q-value ≤ 0.05) (right quadrant in dark red for ECOR and dark blue for ESBL libraries).

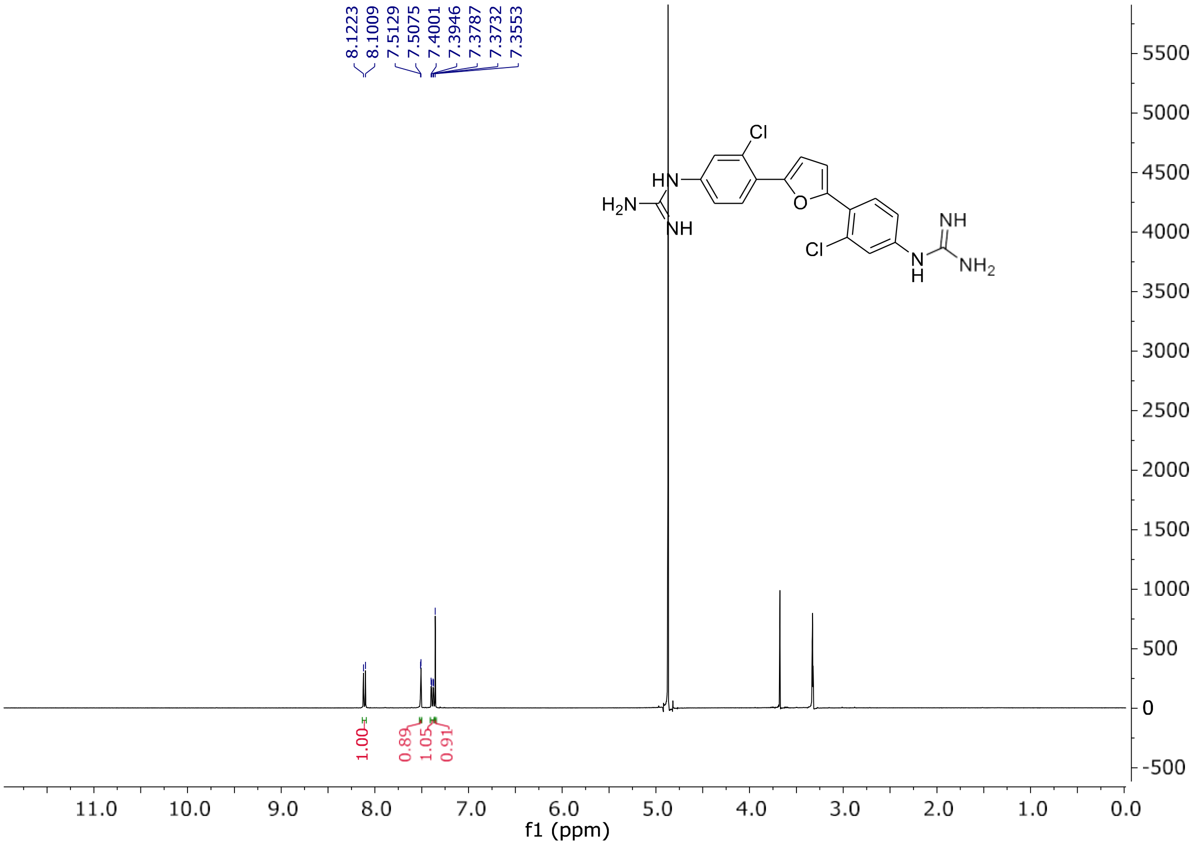
8.1223
8.1009
7.5129
7.5075
7.4001
7.3946
7.3787
7.3732
7.3553
Cl
HN
H2N
NH
O
Cl
NH
N
H
NH2
1.00
0.89
1.05
0.91
f1 (ppm)

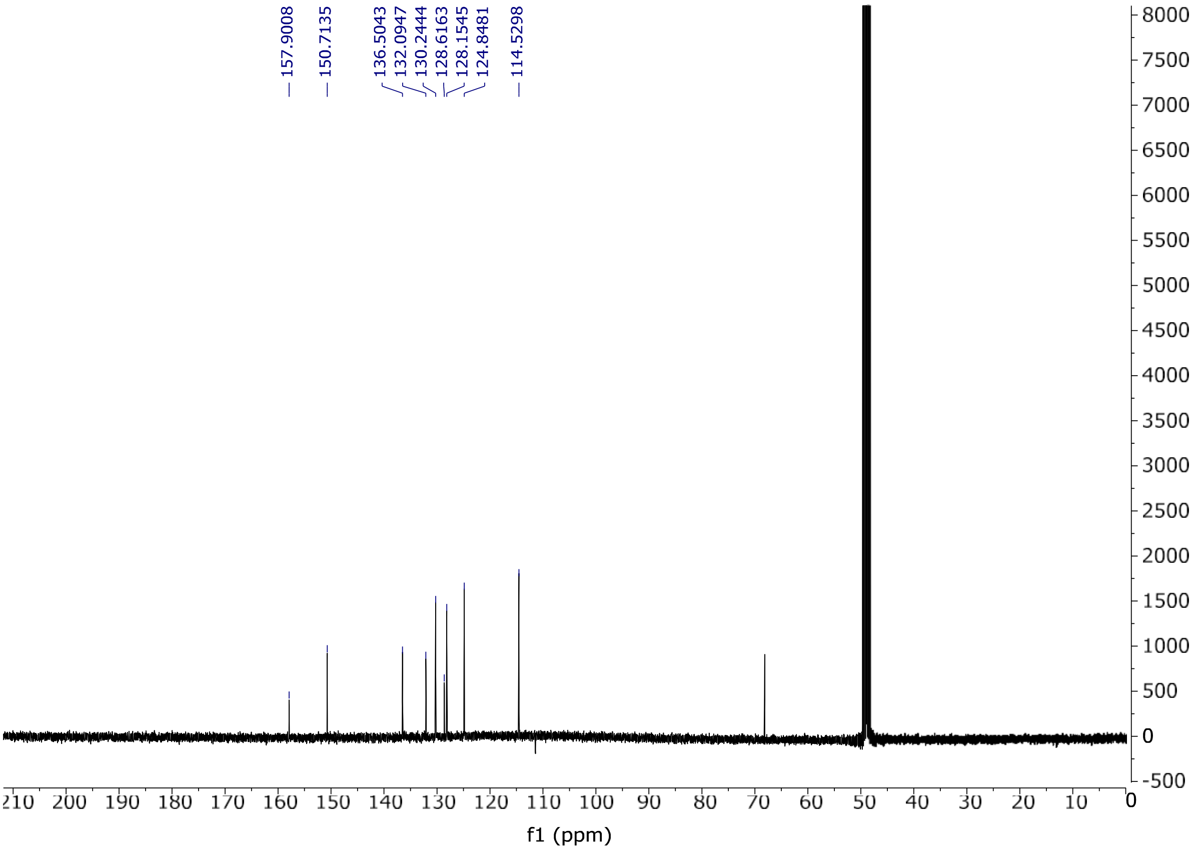
157.9008
150.7135
136.5043
132.0947
130.2444
128.6163
128.1545
124.8481
114.5298
f1 (ppm)

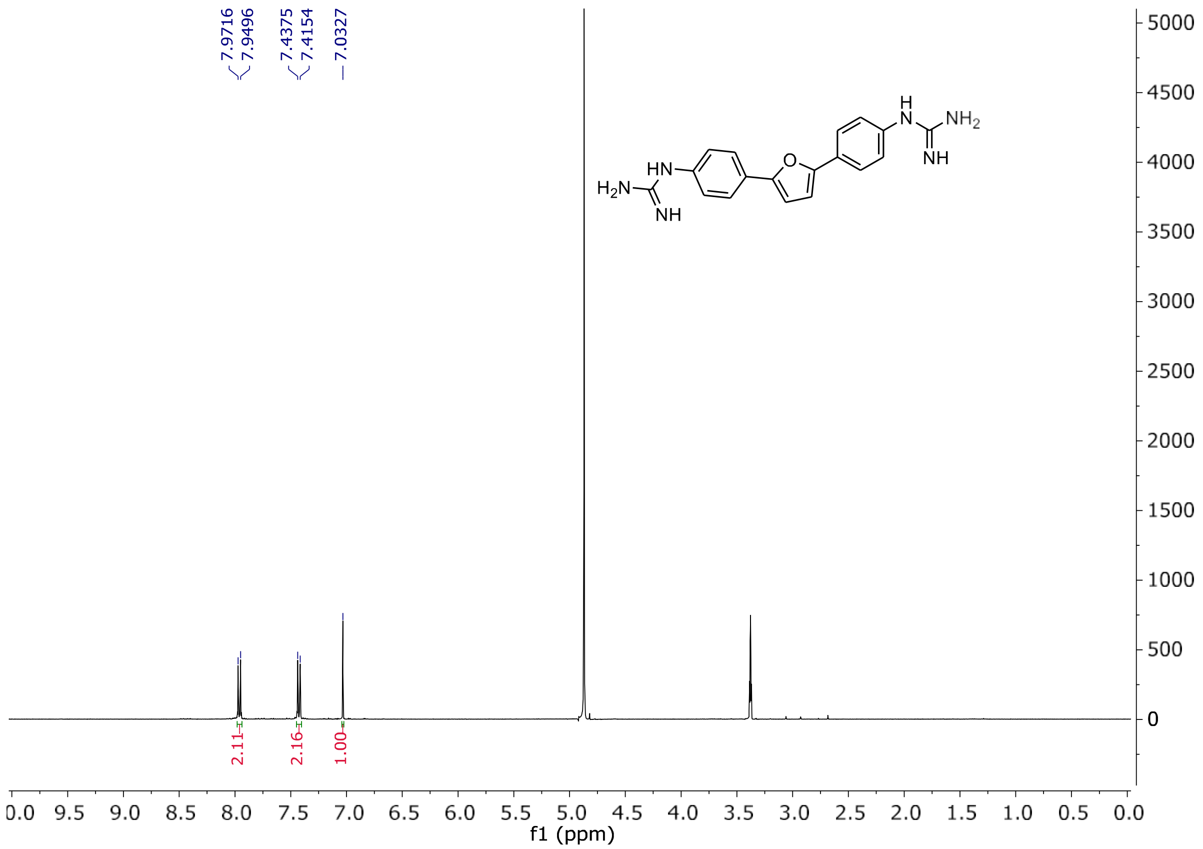

7.9716
7.9496
7.4375
7.4154
7.0327
2.11
2.16
1.00
HN
H2N
NH
O
H
N
NH2
NH
f1 (ppm)

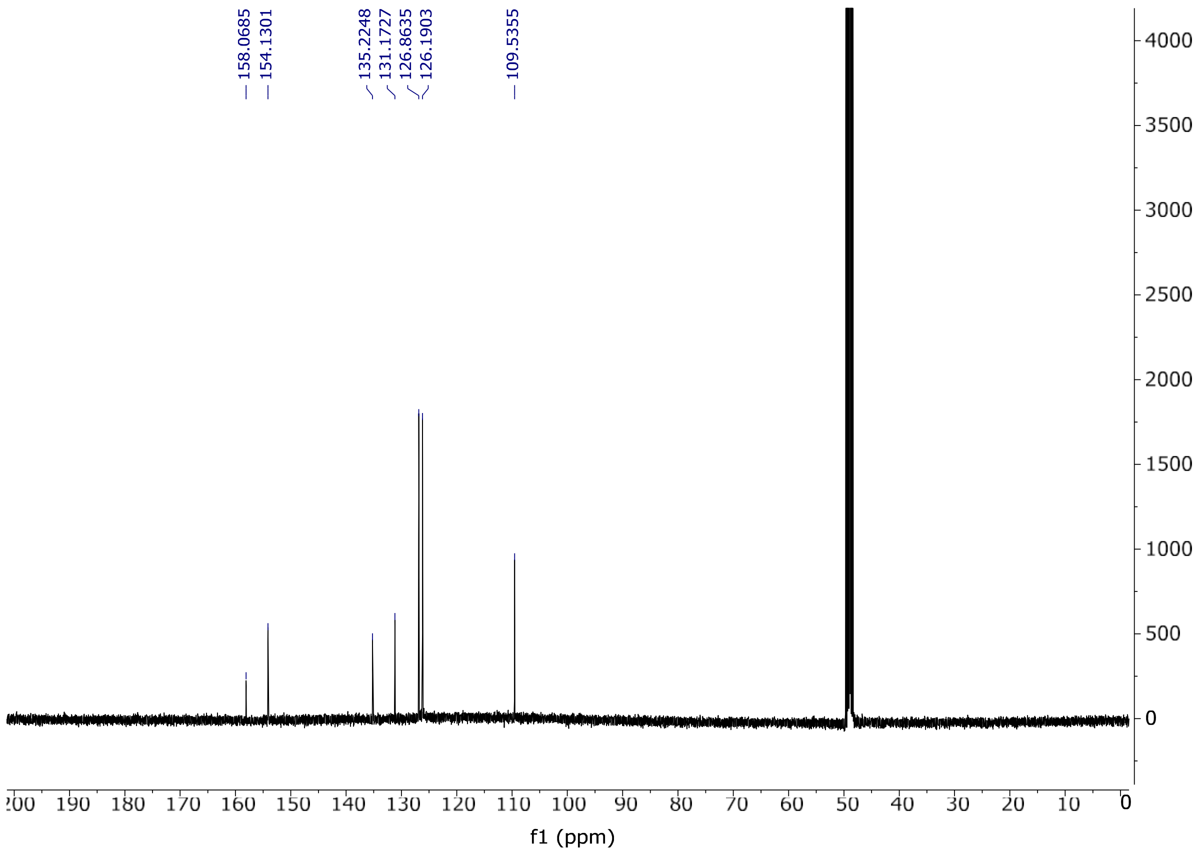

158.0685
154.1301
135.2248
131.1727
126.8635
126.1903
109.5355
f1 (ppm)

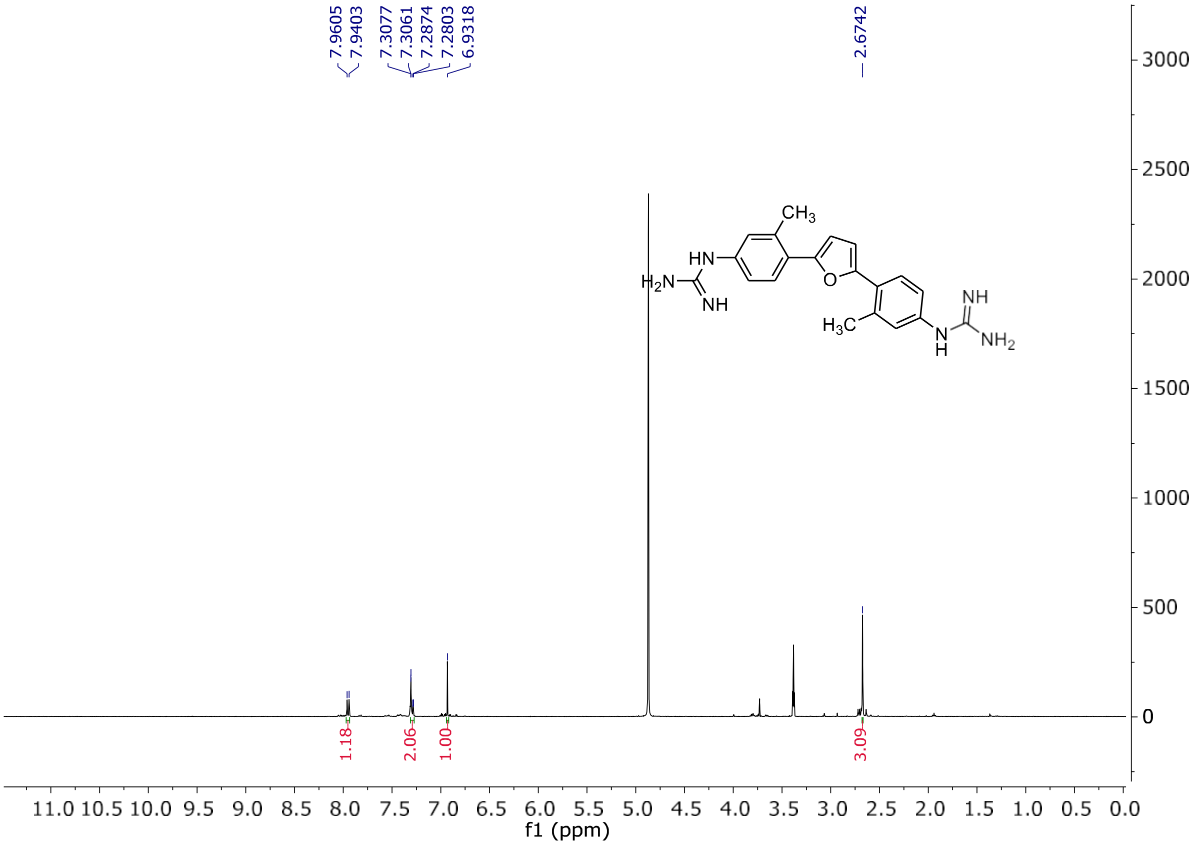
7.9605
7.9403
7.3077
7.3061
7.2874
7.2803
6.9318
2.6742
CH3
HN
H2N
NH
O
H3C
NH
N
H
NH2
1.18
2.06
1.00
3.09
f1 (ppm)

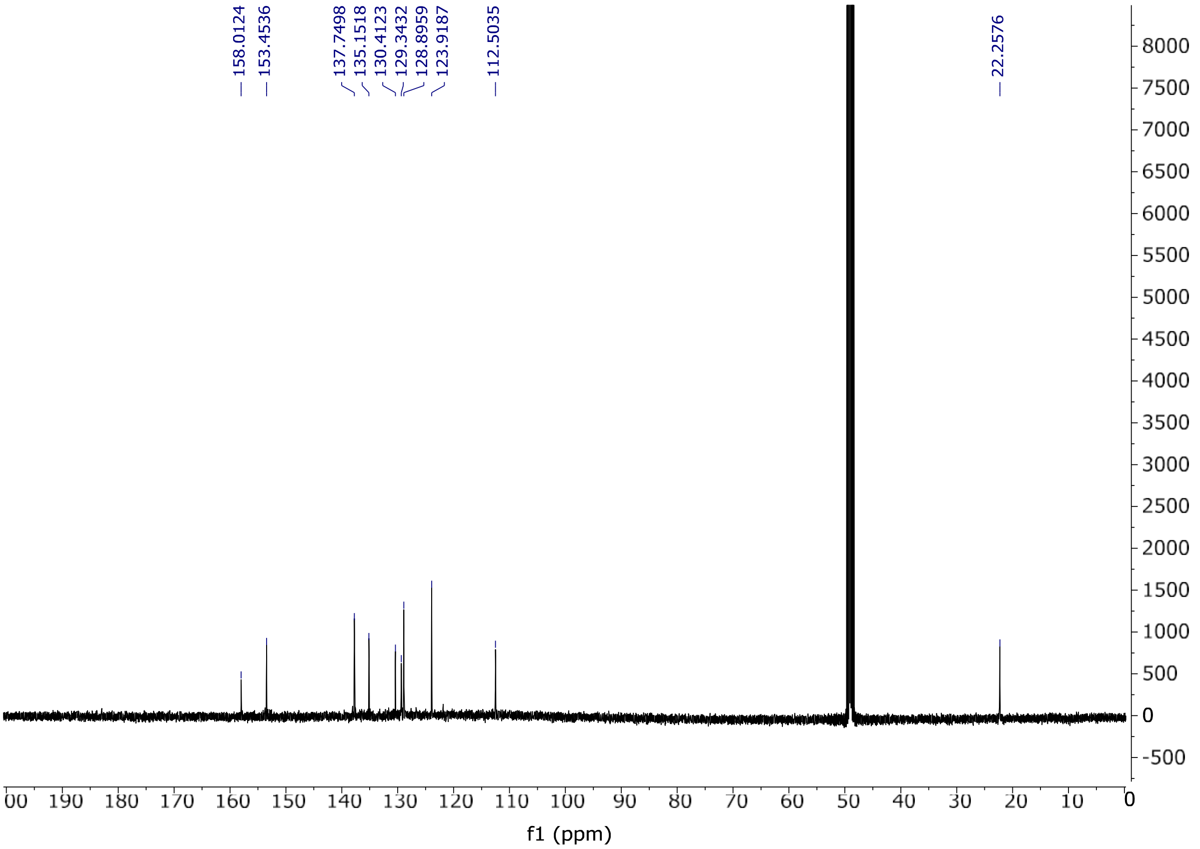
158.0124
153.4536
137.7498
135.1518
130.4123
129.3432
128.8959
123.9187
112.5035
22.2576
f1 (ppm)

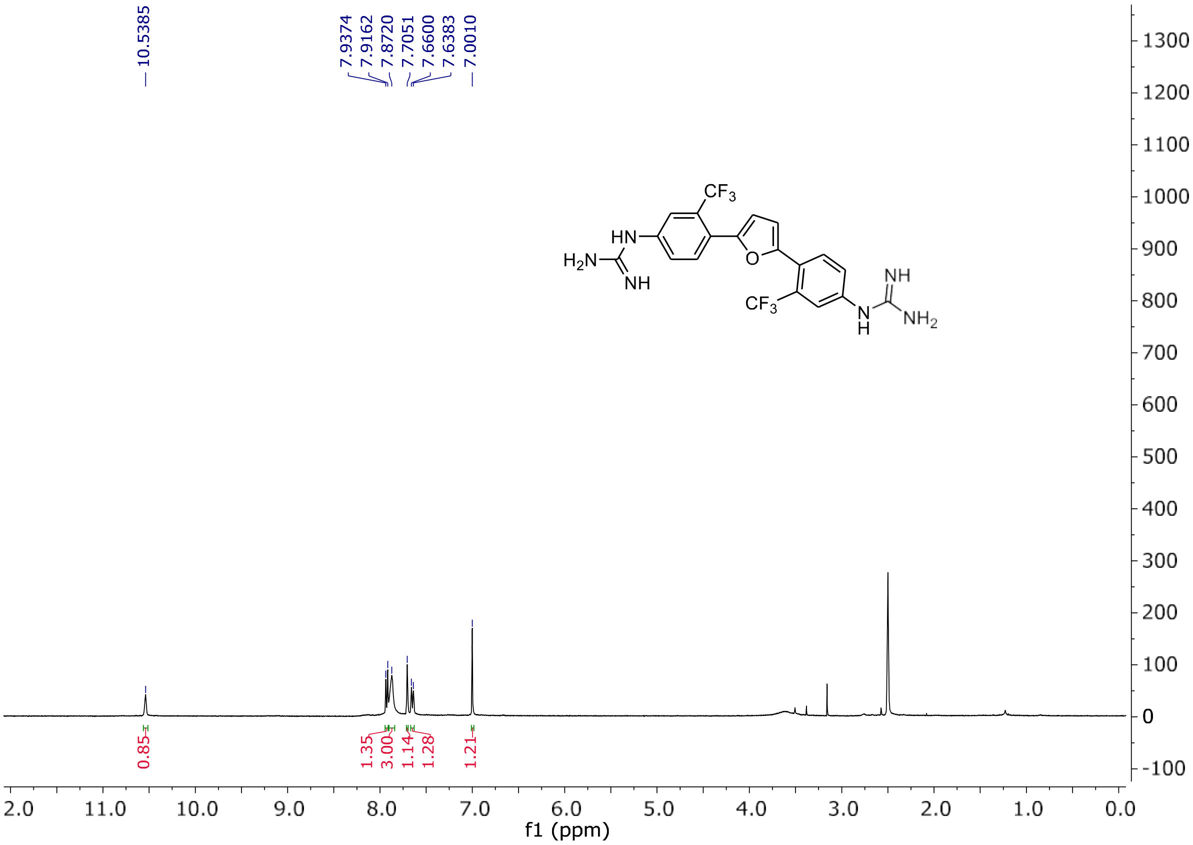
10.5385
7.9374
7.9162
7.8720
7.7051
7.6600
7.6383
7.0010
CF3
HN
H2N
NH
O
CF3
NH
N
H
NH2
0.85
1.35
3.00
1.14
1.28
1.21
f1 (ppm)

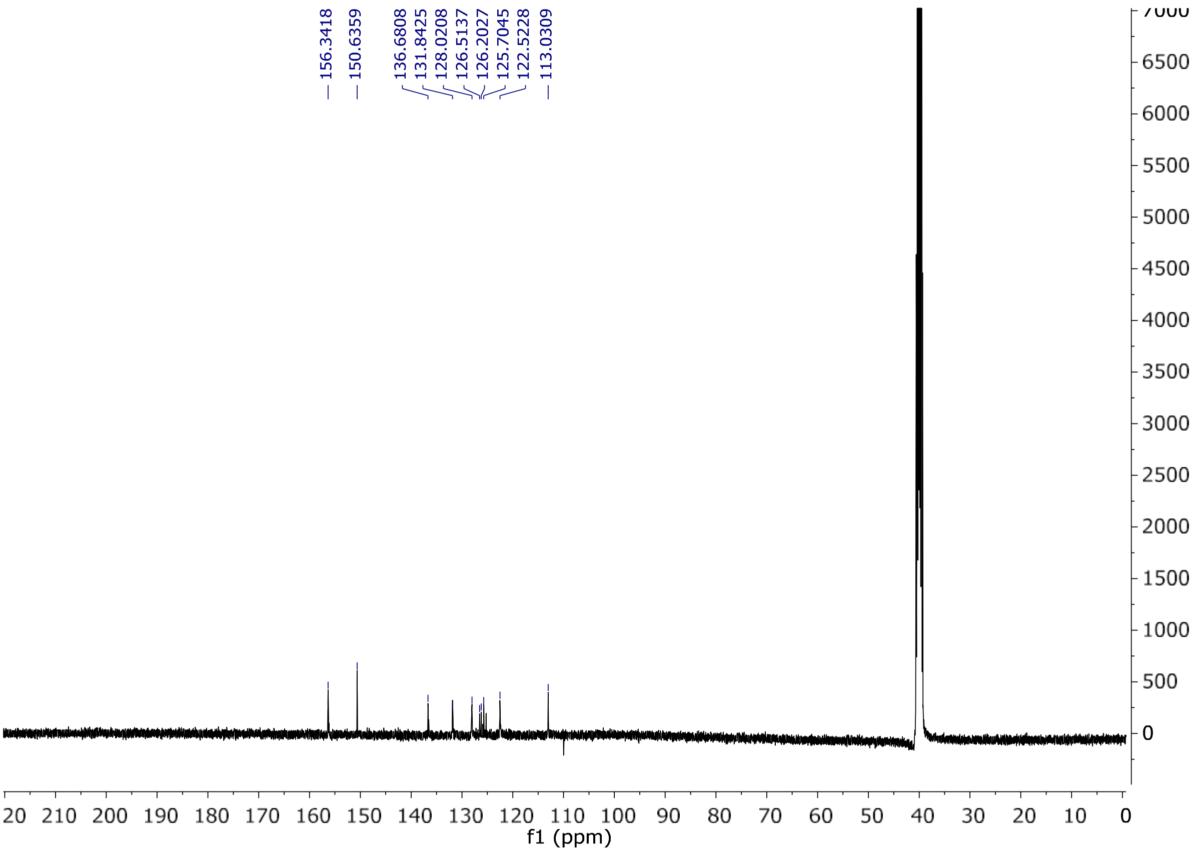
156.3418
150.6359
136.6808
131.8425
128.0208
126.5137
126.2027
125.7045
122.5228
113.0309
f1 (ppm)

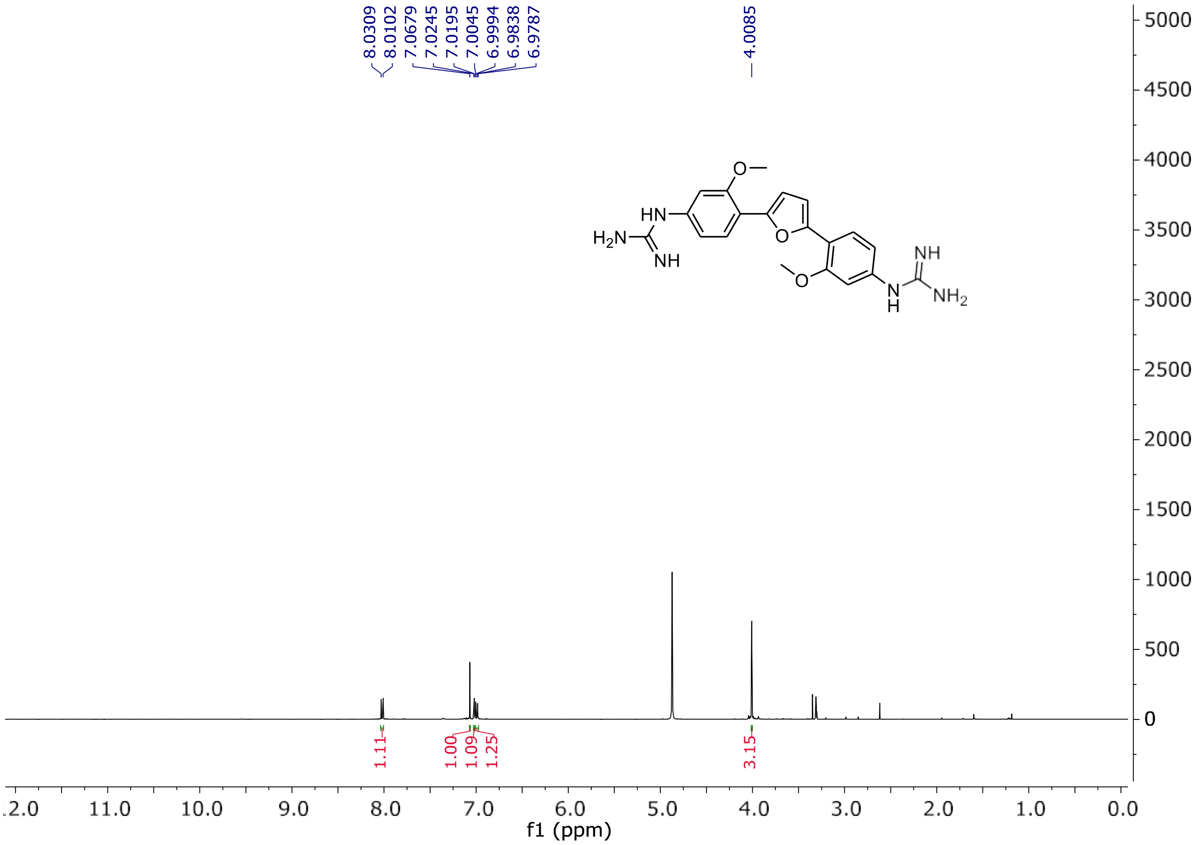
8.0309
8.0102
7.0679
7.0245
7.0195
7.0045
6.9994
6.9838
6.9787
4.0085
1.11
1.00
1.09
1.25
3.15
f1 (ppm)

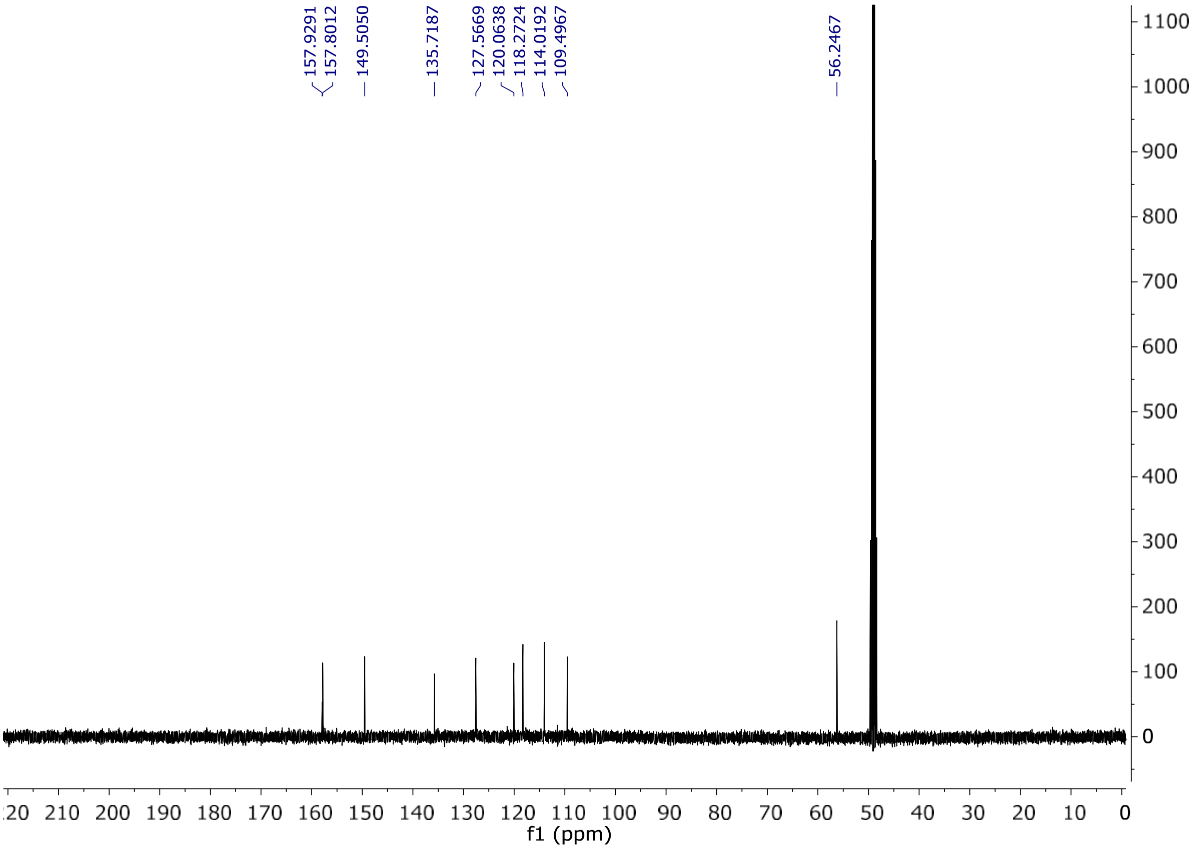
157.9291
157.8012
149.5050
135.7187
127.5669
120.0638
118.2724
114.0192
109.4967
56.2467
f1 (ppm)

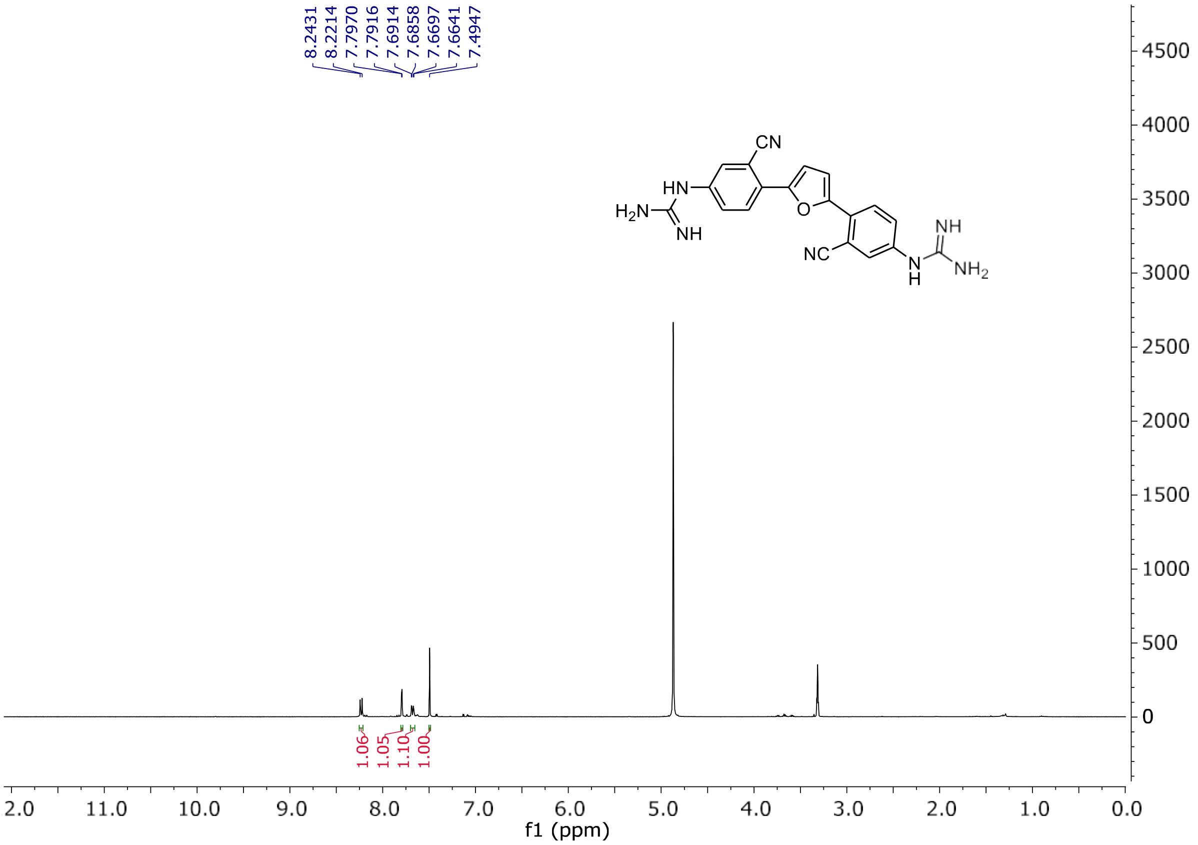
8.2431
8.2214
7.7970
7.7916
7.6914
7.6858
7.6697
7.6641
7.4947
CN
HN
H2N
NH
O
NC
NH
N
H
NH2
1.06
1.05
1.10
1.00
f1 (ppm)

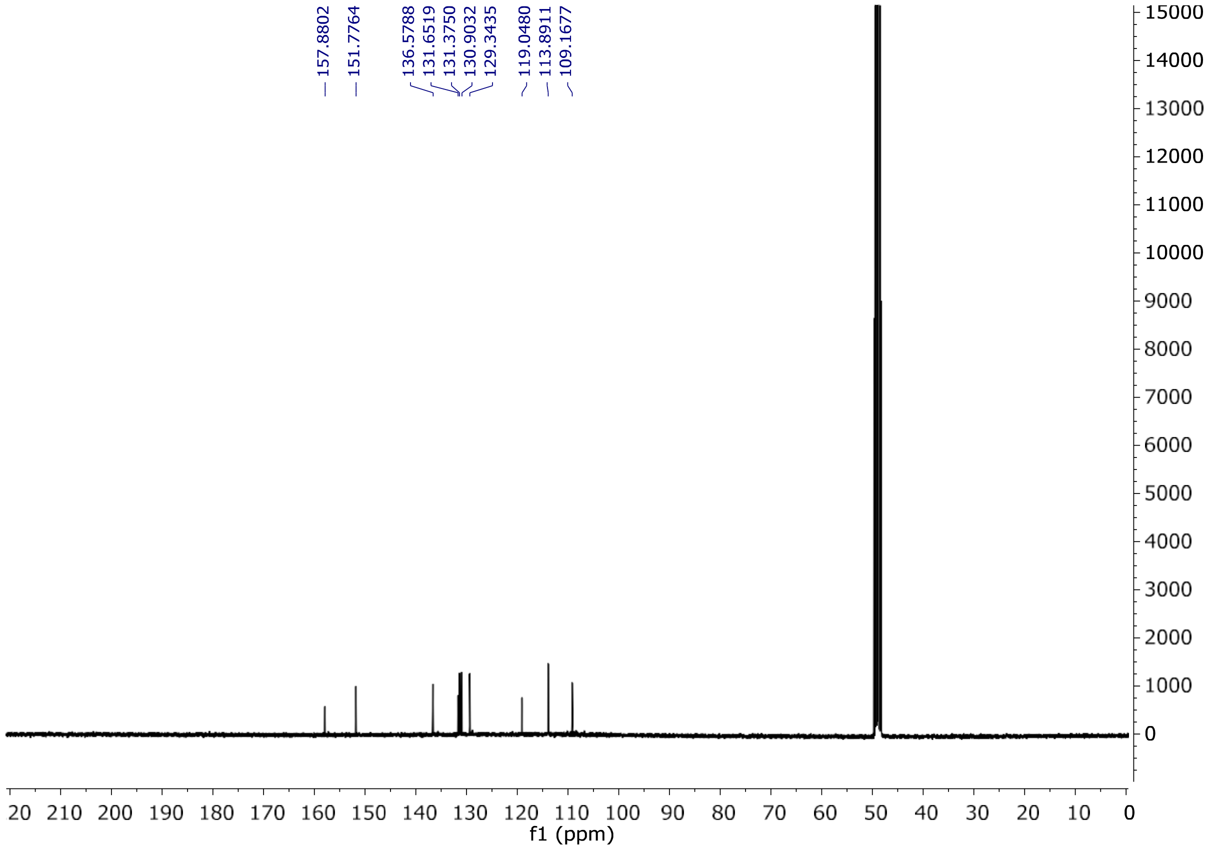
157.8802
151.7764
136.5788
131.6519
131.3750
130.9032
129.3435
119.0480
113.8911
109.1677
f1 (ppm)

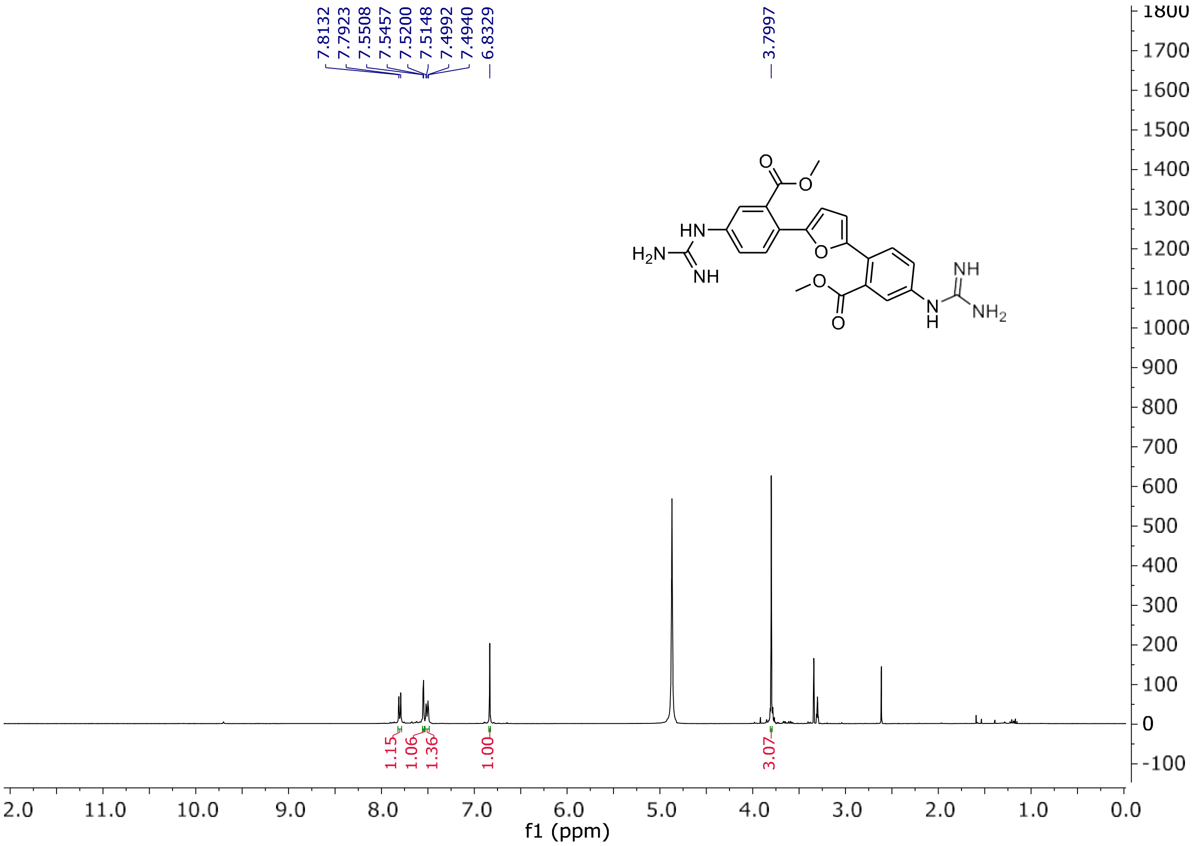
7.8132
7.7923
7.5508
7.5457
7.5200
7.5148
7.4992
7.4940
6.8329
3.7997
HN
H2N
NH
O
O
O
O
O
NH
N
H
NH2
1.15
1.06
1.36
1.00
3.07
2.0 11.0 10.0 9.0 8.0 7.0 6.0 5.0 4.0 3.0 2.0 1.0 0.0
f1 (ppm)
1800 1700 1600 1500 1400 1300 1200 1100 1000 900 800 700 600 500 400 300 200 100 0 -100

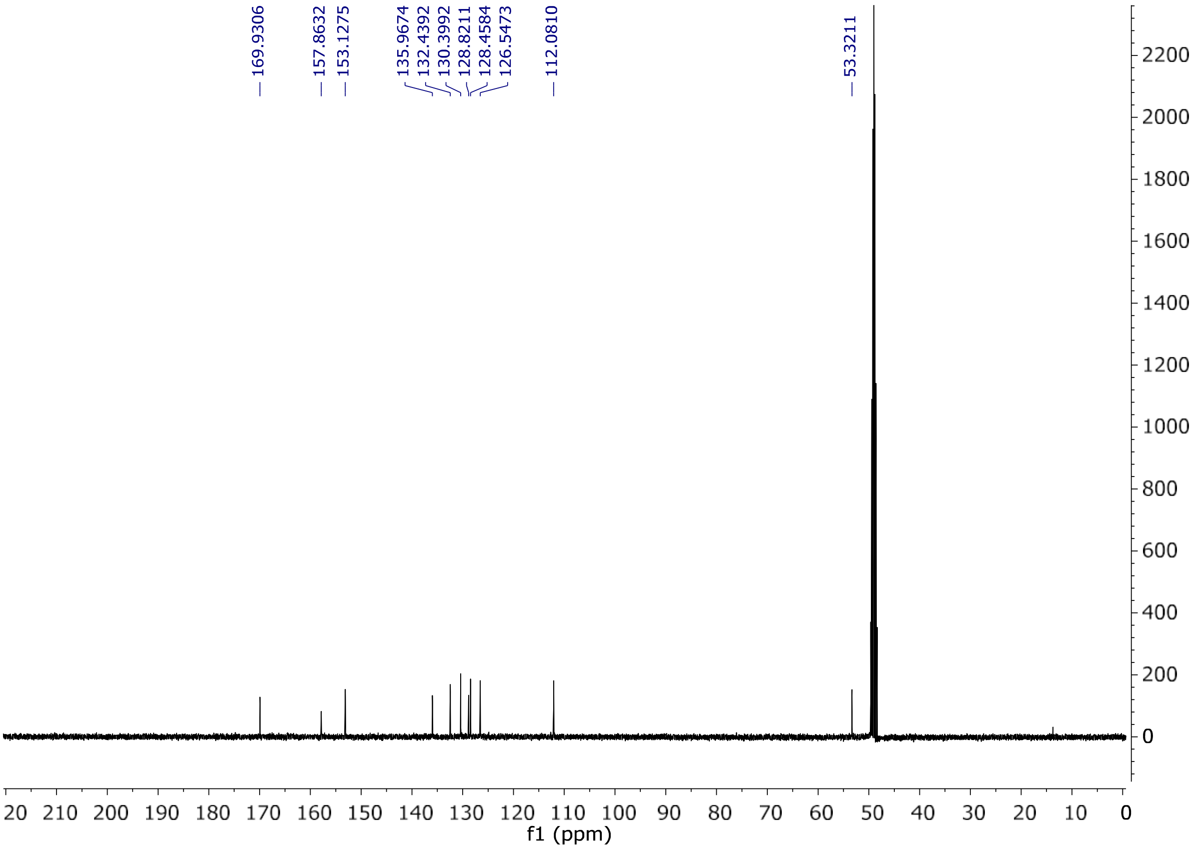
169.9306
157.8632
153.1275
135.9674
132.4392
130.3992
128.8211
128.4584
126.5473
112.0810
53.3211
20 210 200 190 180 170 160 150 140 130 120 110 100 90 80 70 60 50 40 30 20 10 0
f1 (ppm)
2200 2000 1800 1600 1400 1200 1000 800 600 400 200 0

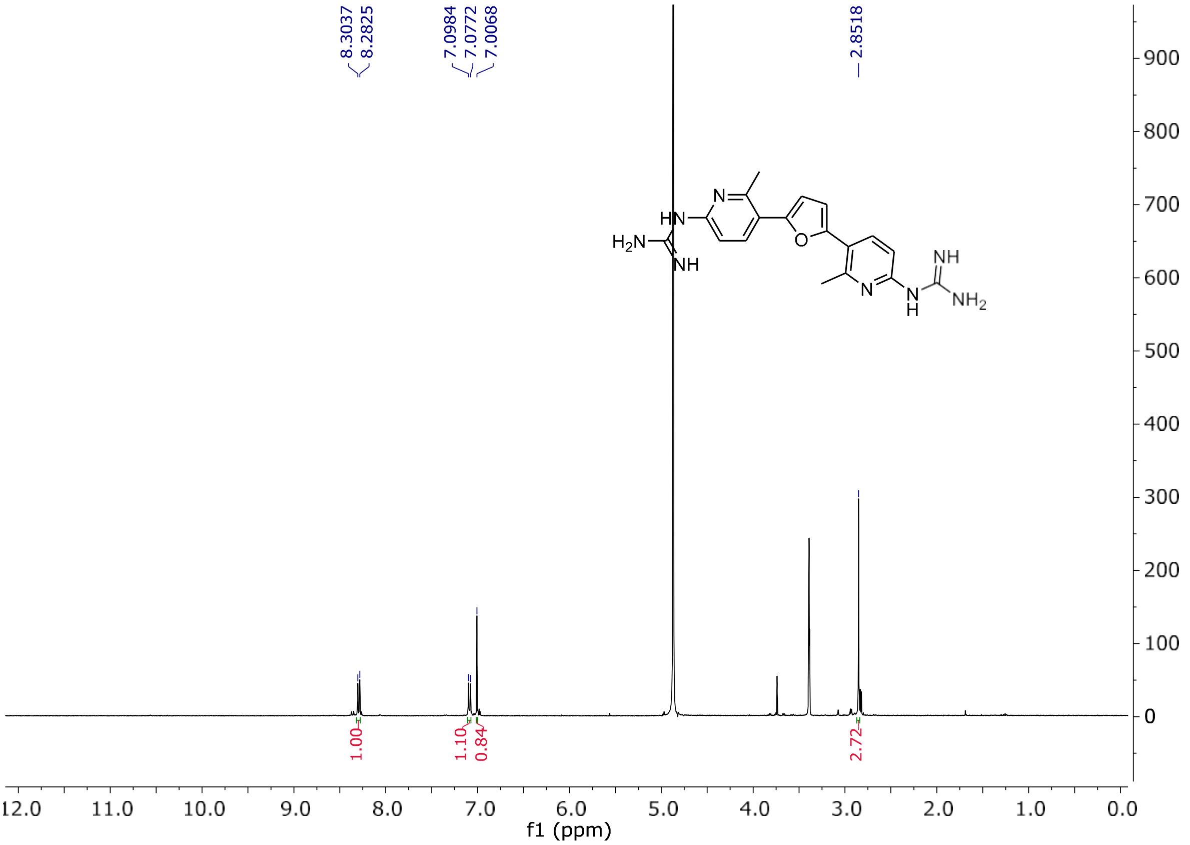

8.3037
8.2825
7.0984
7.0772
7.0068
2.8518
HN
N
H2N
NH
O
N
N
H
NH
NH2
1.00
1.10
0.84
2.72
900
800
700
600
500
400
300
200
100
0
12.0 11.0 10.0 9.0 8.0 7.0 6.0 5.0 4.0 3.0 2.0 1.0 0.0
f1 (ppm)

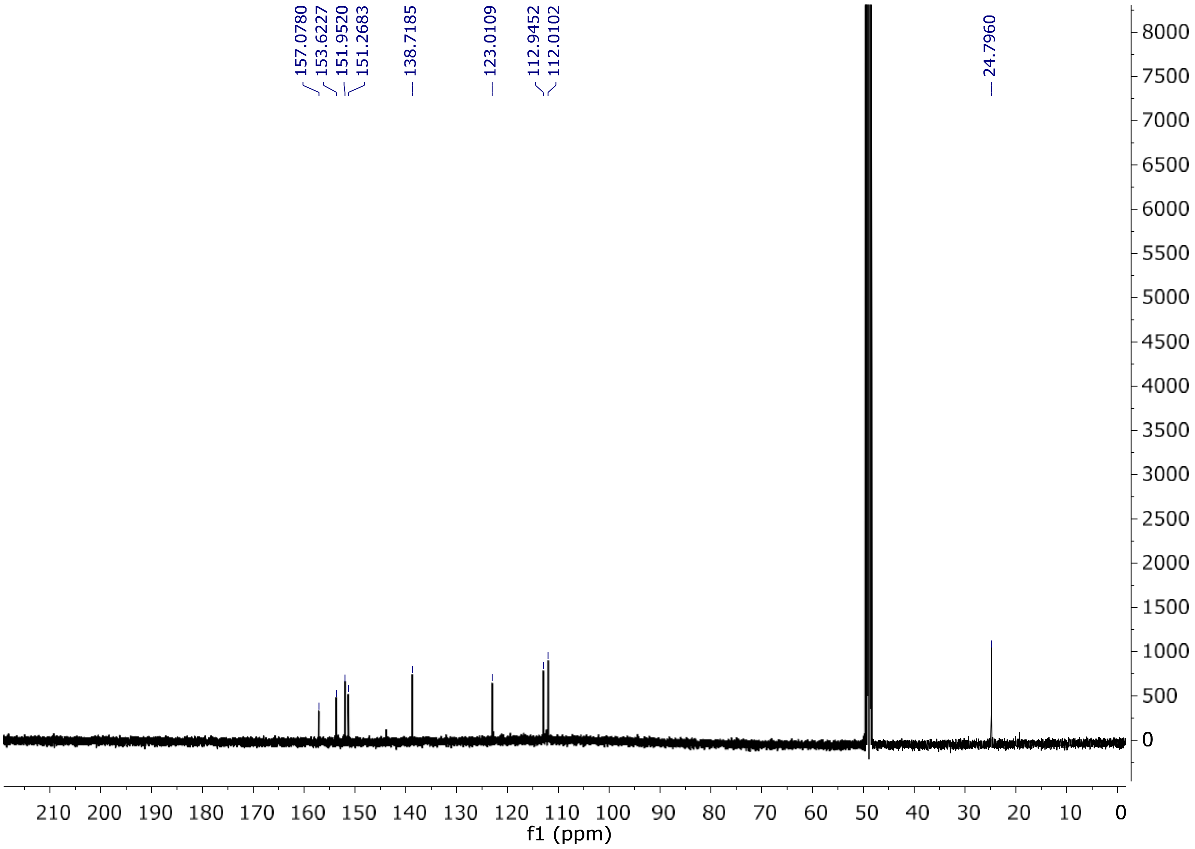

157.0780
153.6227
151.9520
151.2683
138.7185
123.0109
112.9452
112.0102
24.7960
8000
7500
7000
6500
6000
5500
5000
4500
4000
3500
3000
2500
2000
1500
1000
500
0
210 200 190 180 170 160 150 140 130 120 110 100 90 80 70 60 50 40 30 20 10 0
f1 (ppm)

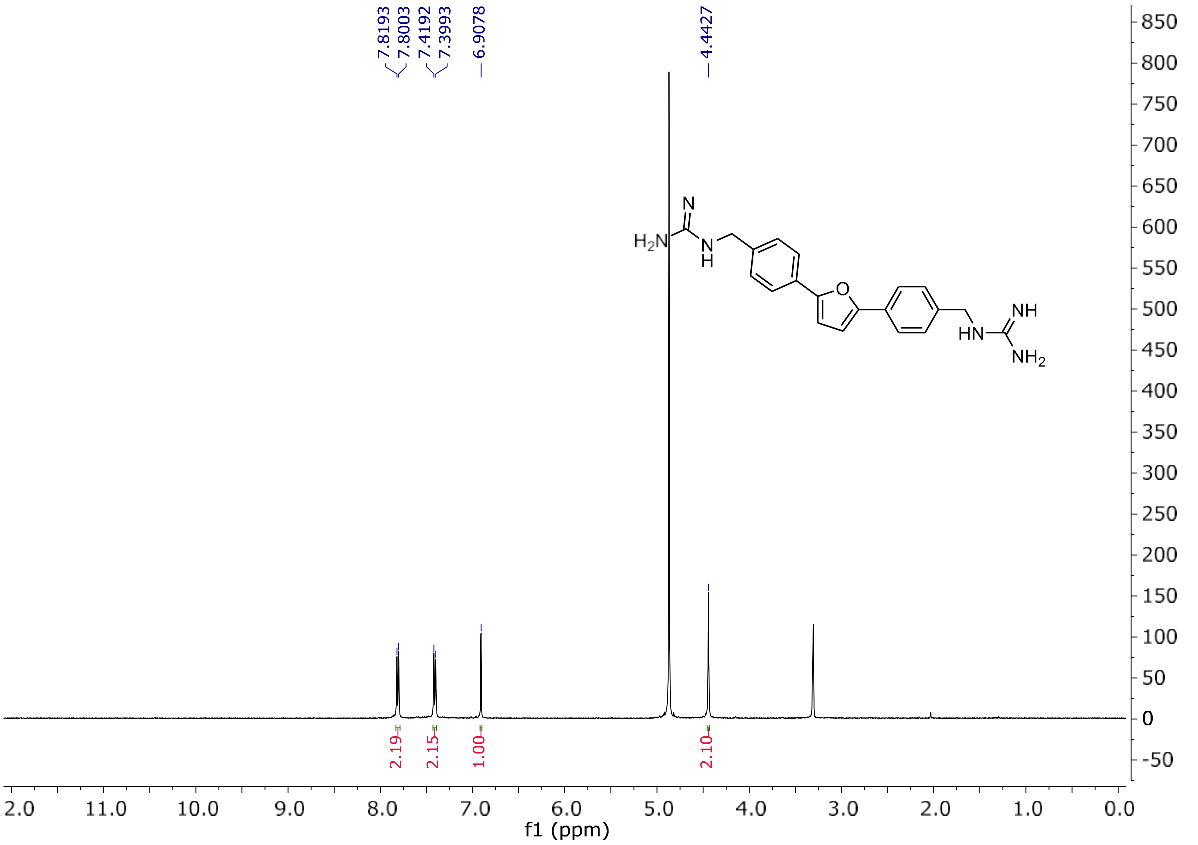
7.8193
7.8003
7.4192
7.3993
6.9078
4.4427
2.19
2.15
1.00
2.10
H_2N
N
N
H
O
NH
HN
NH_2
f1 (ppm)

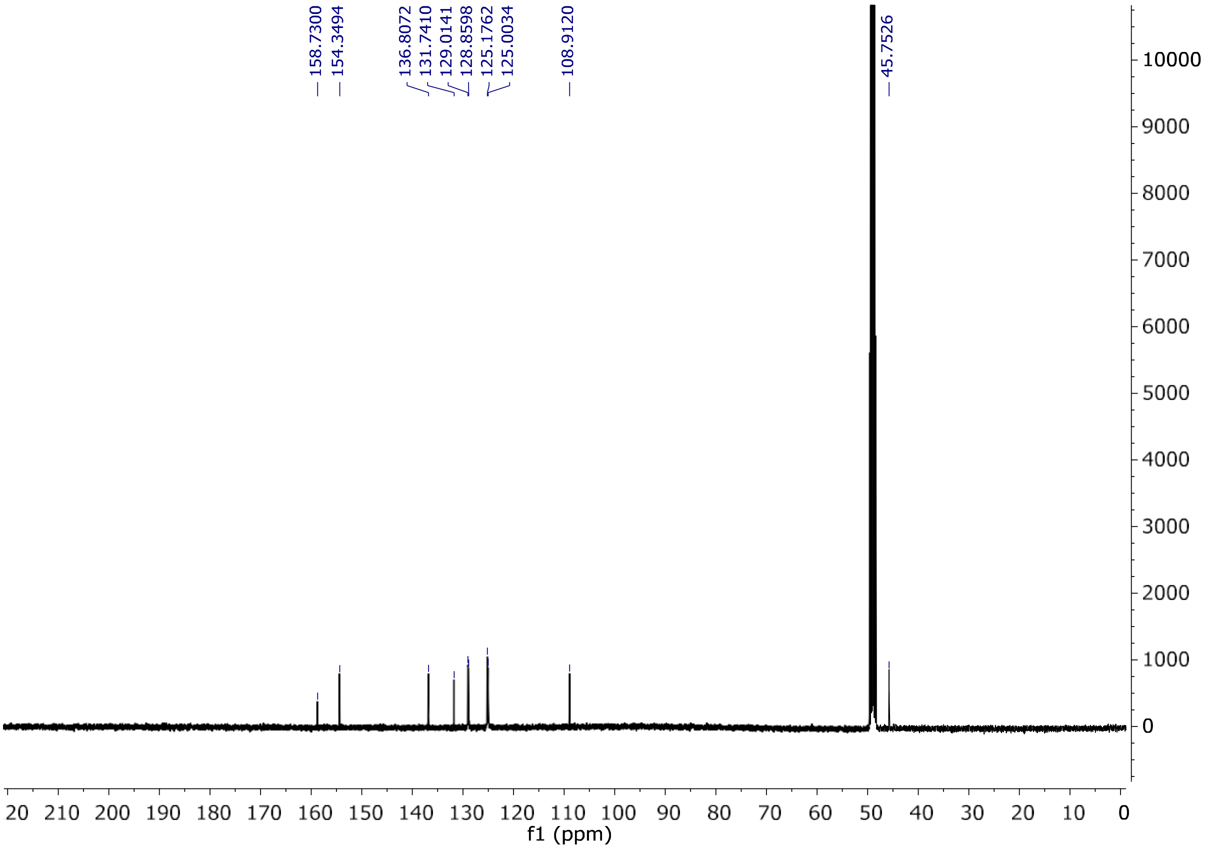
158.7300
154.3494
136.8072
131.7410
129.0141
128.8598
125.1762
125.0034
108.9120
45.7526
f1 (ppm)

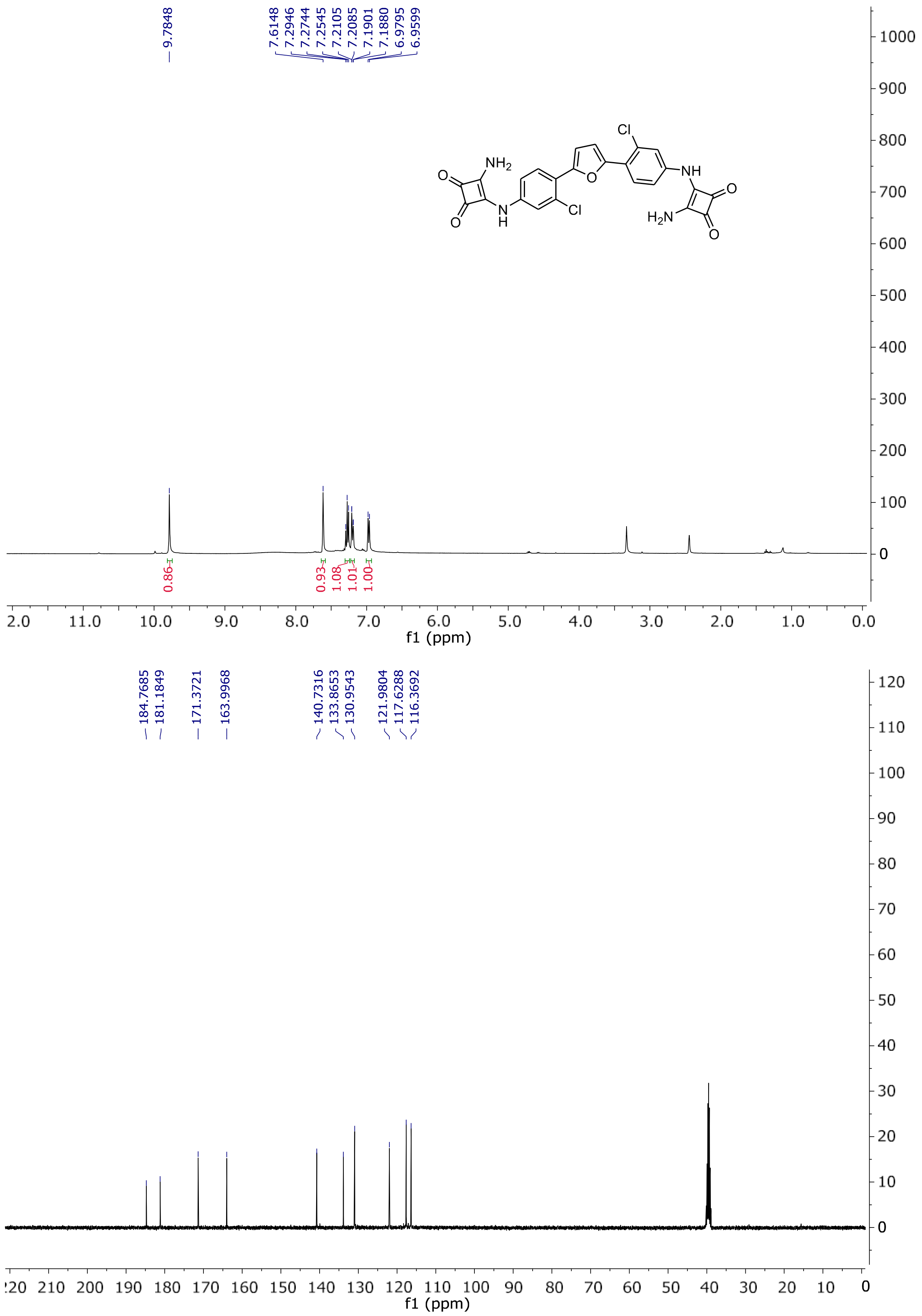

Figure S24. ^{1}H NMR (DMSO-d$_6$, 400 MHz) and ^{13}C NMR (DMSO-d$_6$, 100 MHz) spectra for 2,5-Bis(2-chloro-4-((3,4-Dioxo-2-(amino)cyclobut-1-en-1-yl)amino)phenyl)furan (**11**).

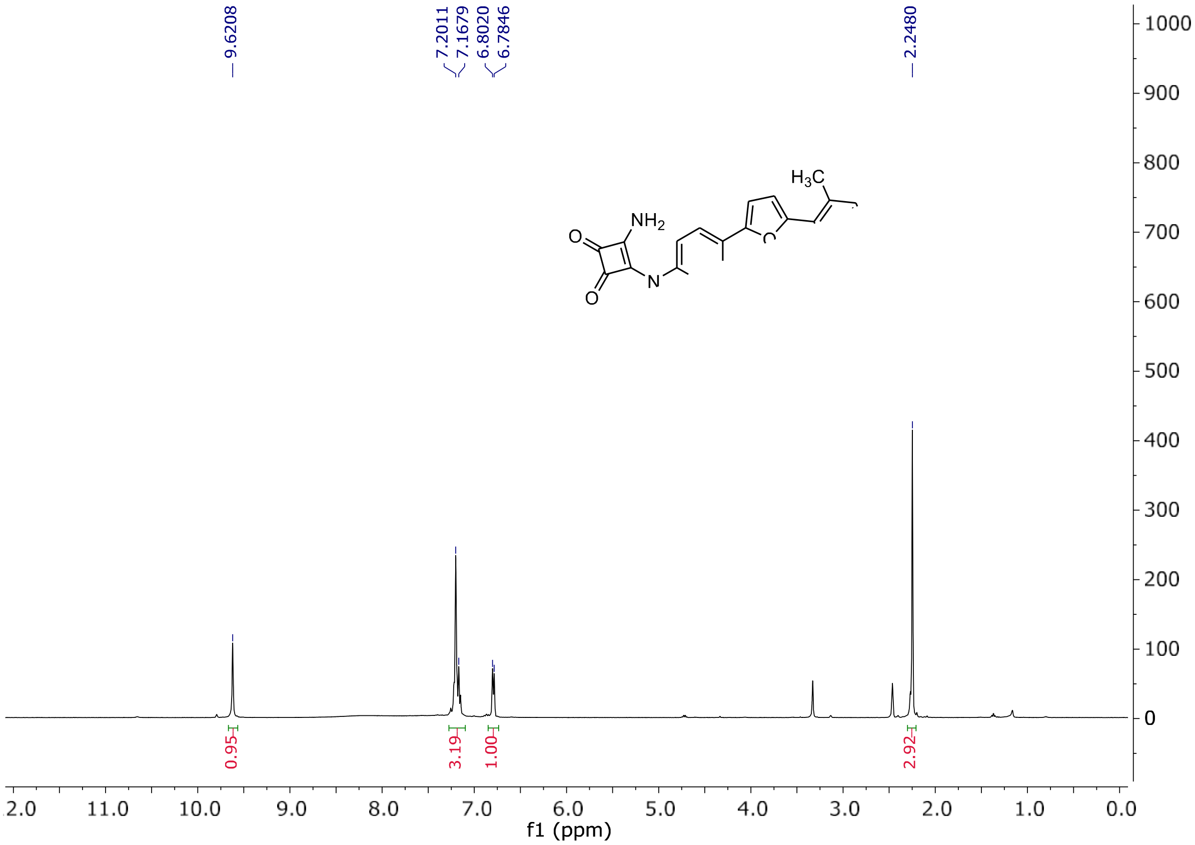
9.6208
7.2011
7.1679
6.8020
6.7846
2.2480
NH2
O
O
N
O
H3C
0.95
3.19
1.00
2.92
f1 (ppm)

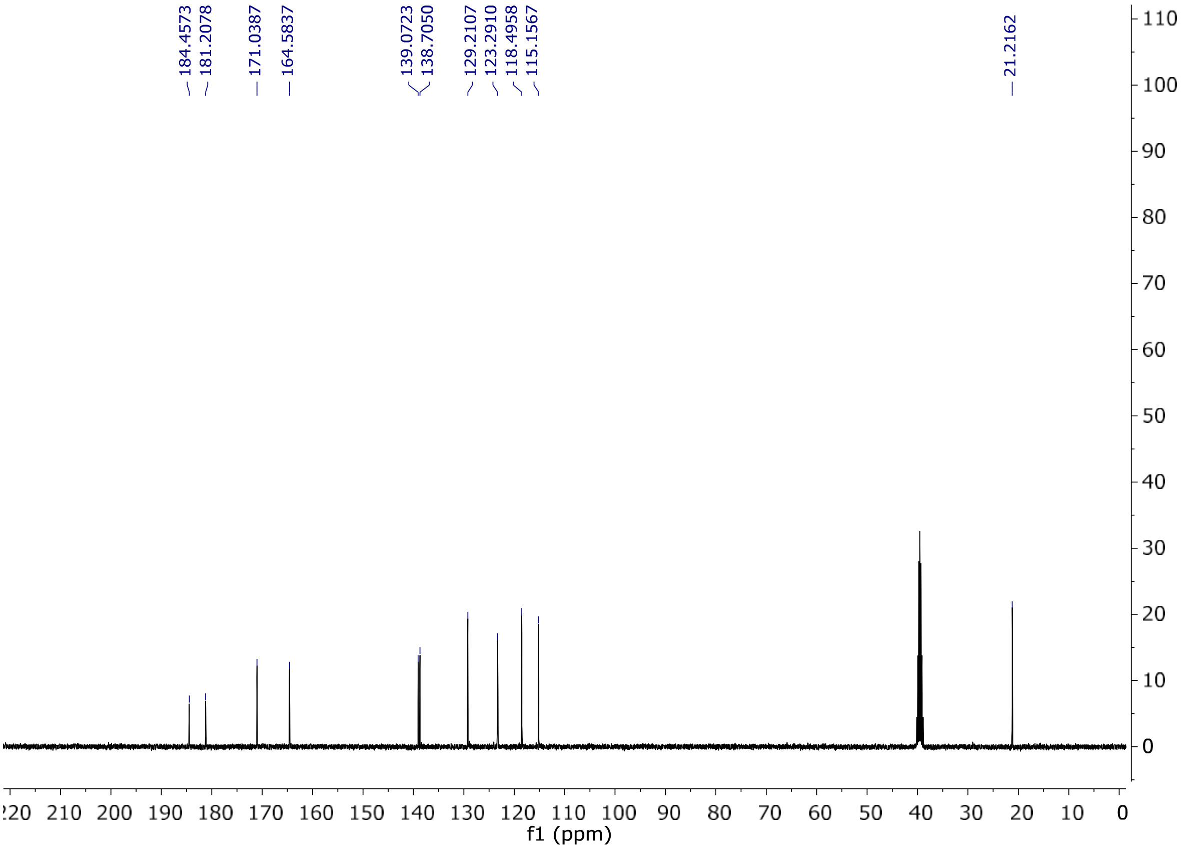
184.4573
181.2078
171.0387
164.5837
139.0723
138.7050
129.2107
123.2910
118.4958
115.1567
21.2162
f1 (ppm)

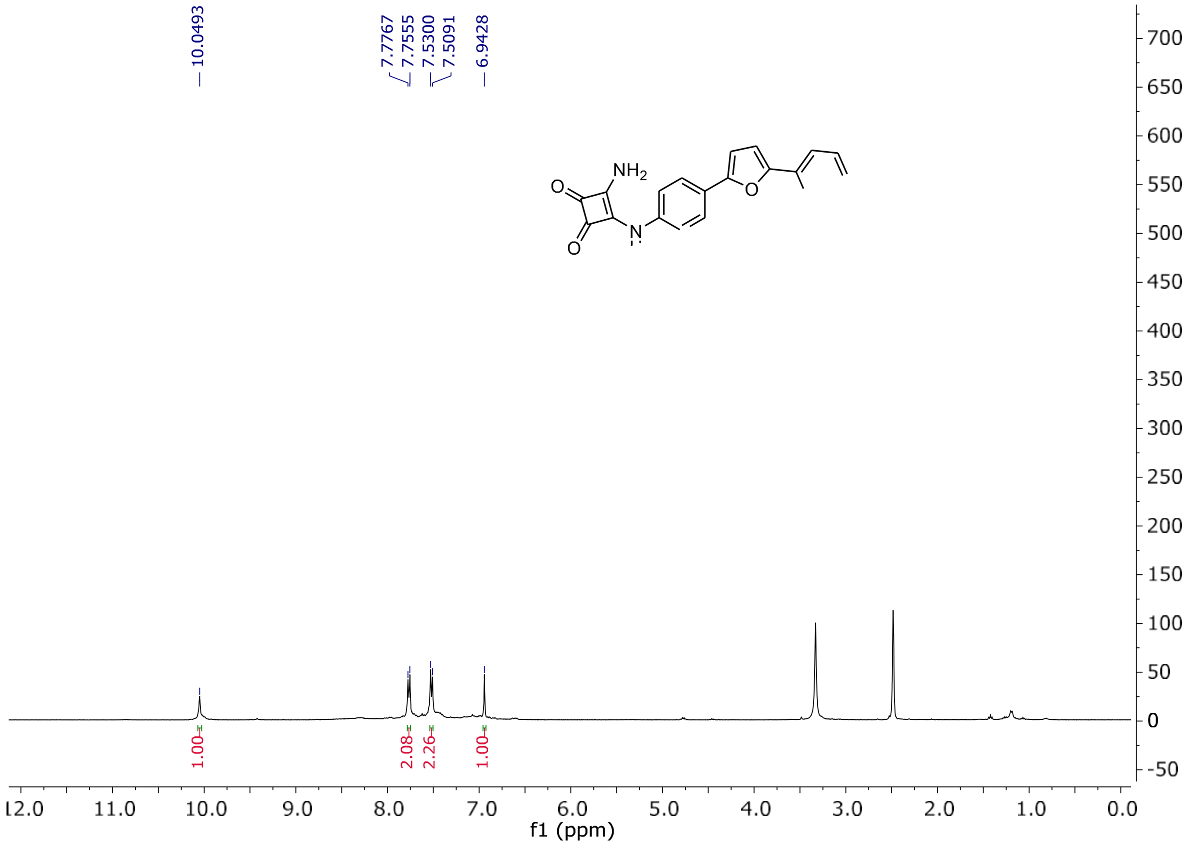

10.0493
7.7767
7.7555
7.5300
7.5091
6.9428
NH2
O
O
N
O
1.00
2.08
2.26
1.00
700
650
600
550
500
450
400
350
300
250
200
150
100
50
0
-50
12.0 11.0 10.0 9.0 8.0 7.0 6.0 5.0 4.0 3.0 2.0 1.0 0.0
f1 (ppm)

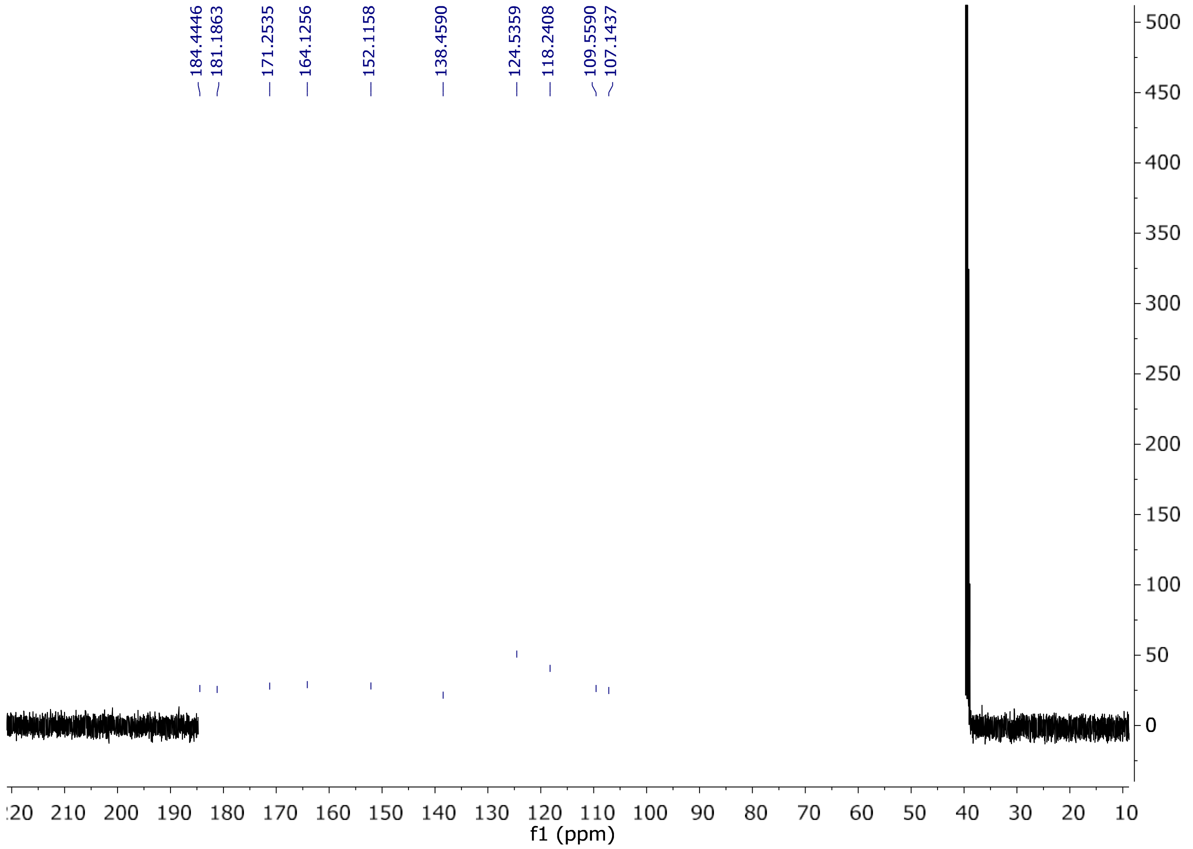

184.4446
181.1863
171.2535
164.1256
152.1158
138.4590
124.5359
118.2408
109.5590
107.1437
500
450
400
350
300
250
200
150
100
50
0
220 210 200 190 180 170 160 150 140 130 120 110 100 90 80 70 60 50 40 30 20 10
f1 (ppm)

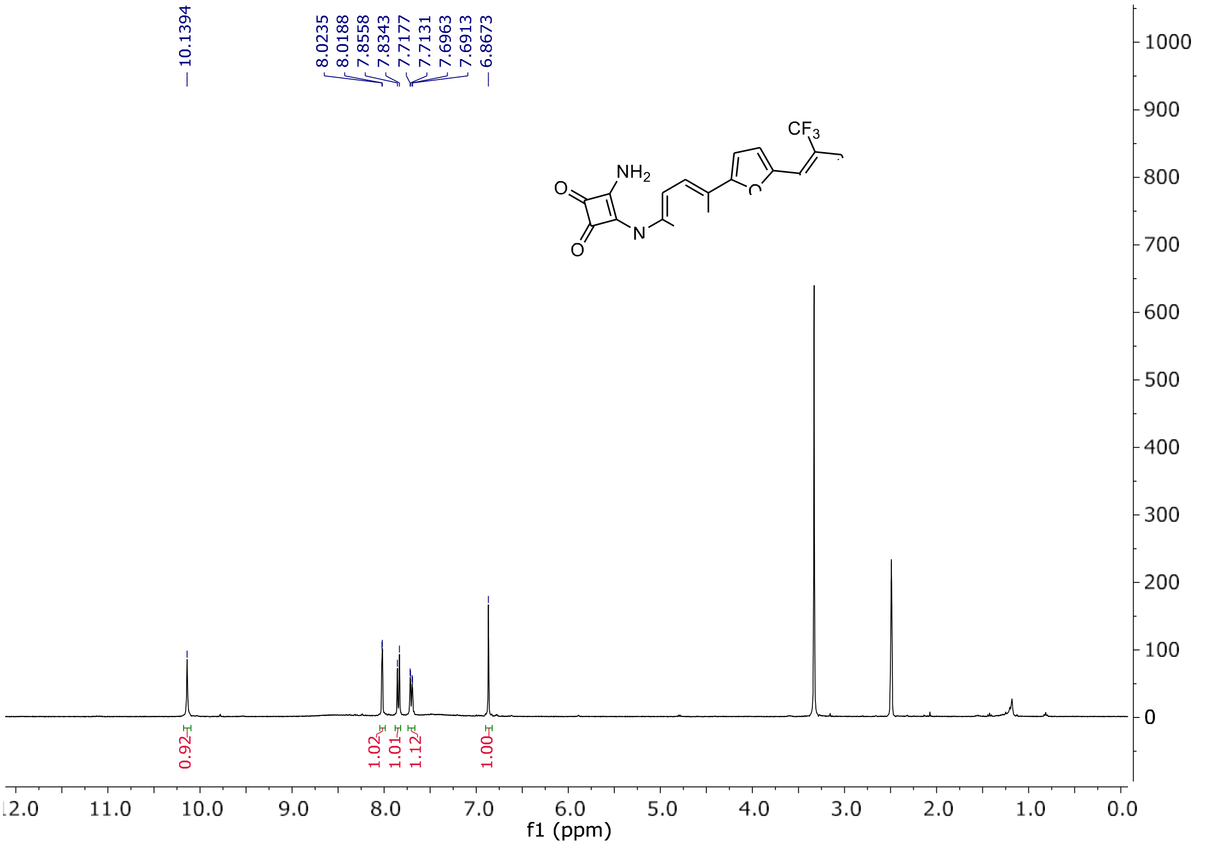
10.1394
8.0235
8.0188
7.8558
7.8343
7.7177
7.7131
7.6963
7.6913
6.8673
CF3
NH2
O
N
0.92
1.02
1.01
1.12
1.00
f1 (ppm)

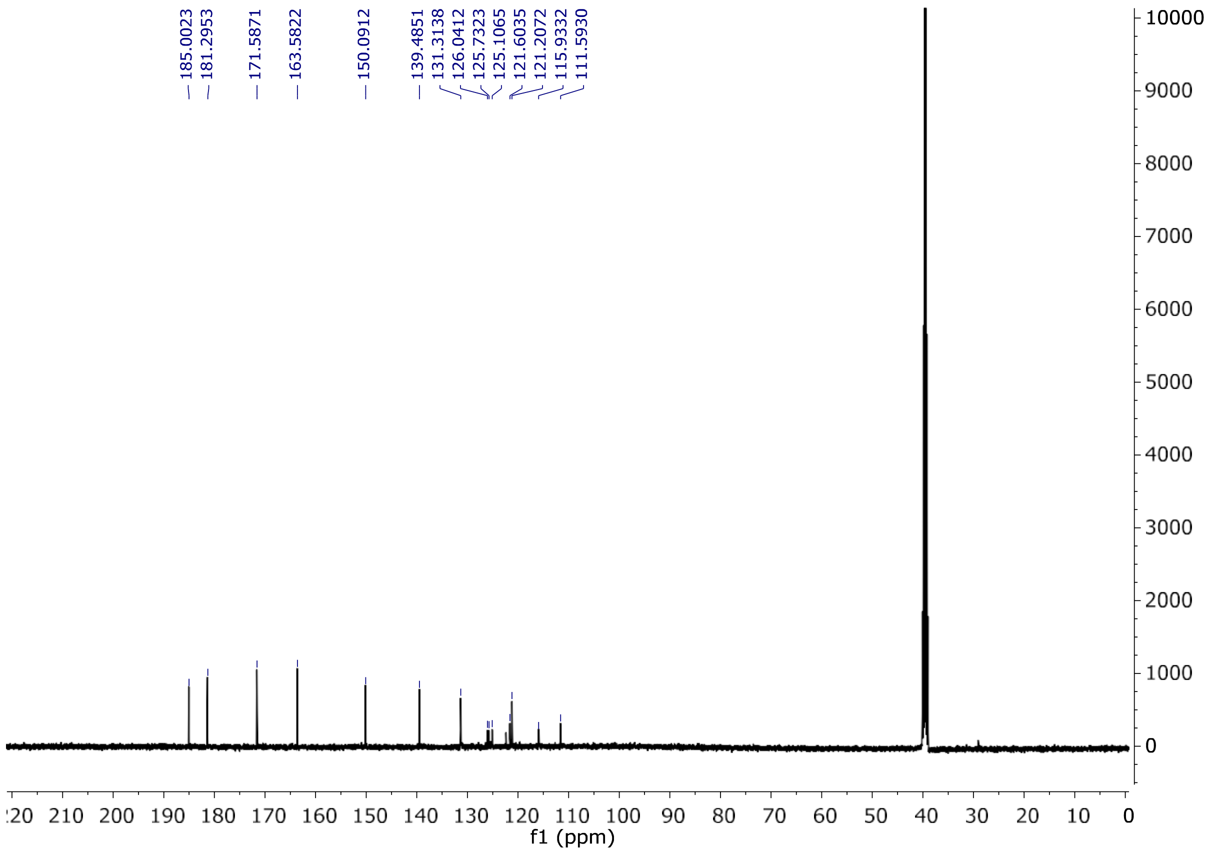
185.0023
181.2953
171.5871
163.5822
150.0912
139.4851
131.3138
126.0412
125.7323
125.1065
121.6035
121.2072
115.9332
111.5930
f1 (ppm)

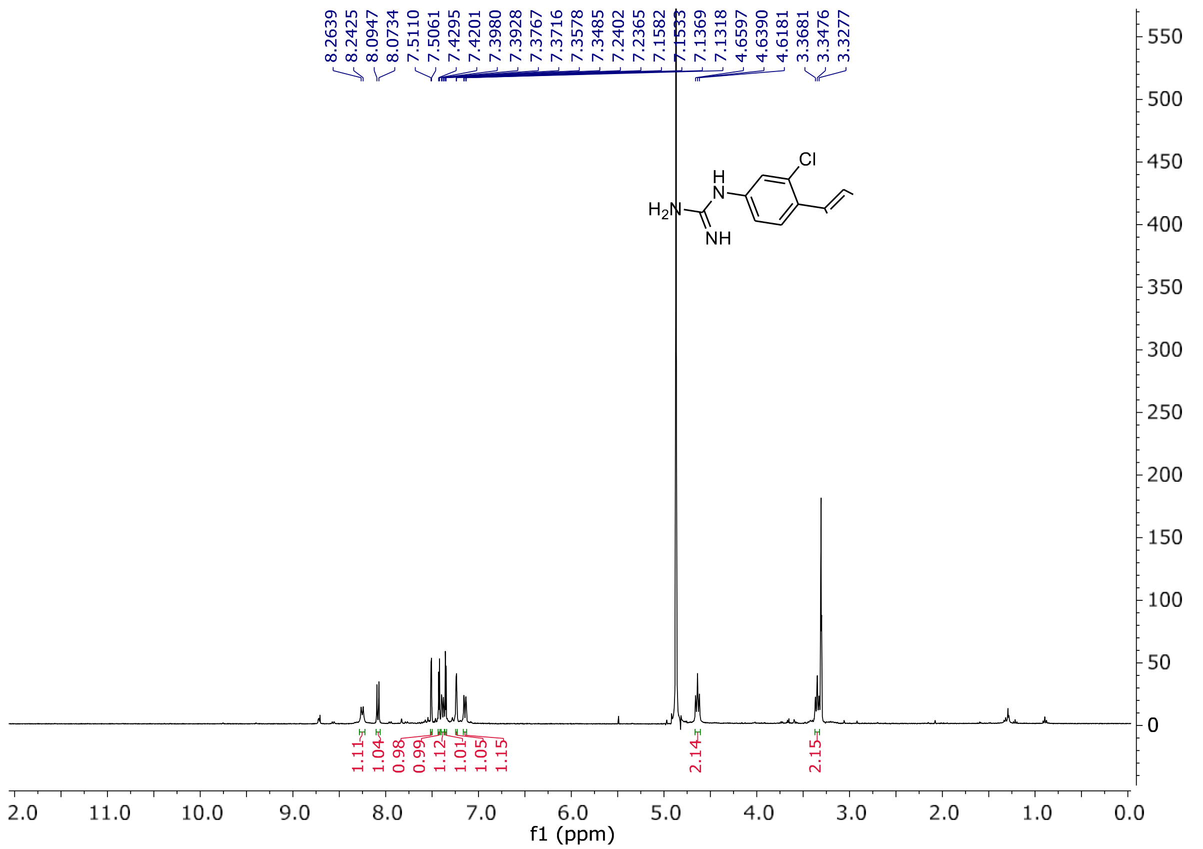

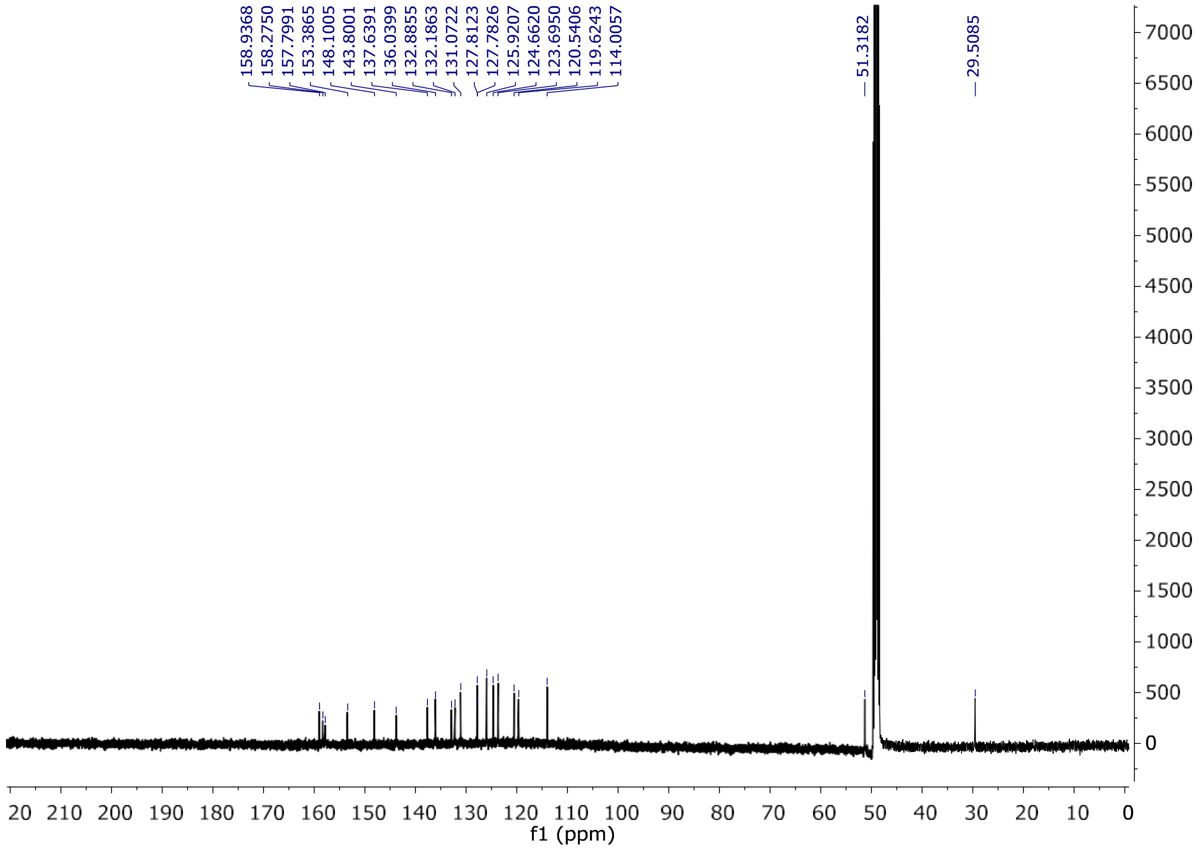

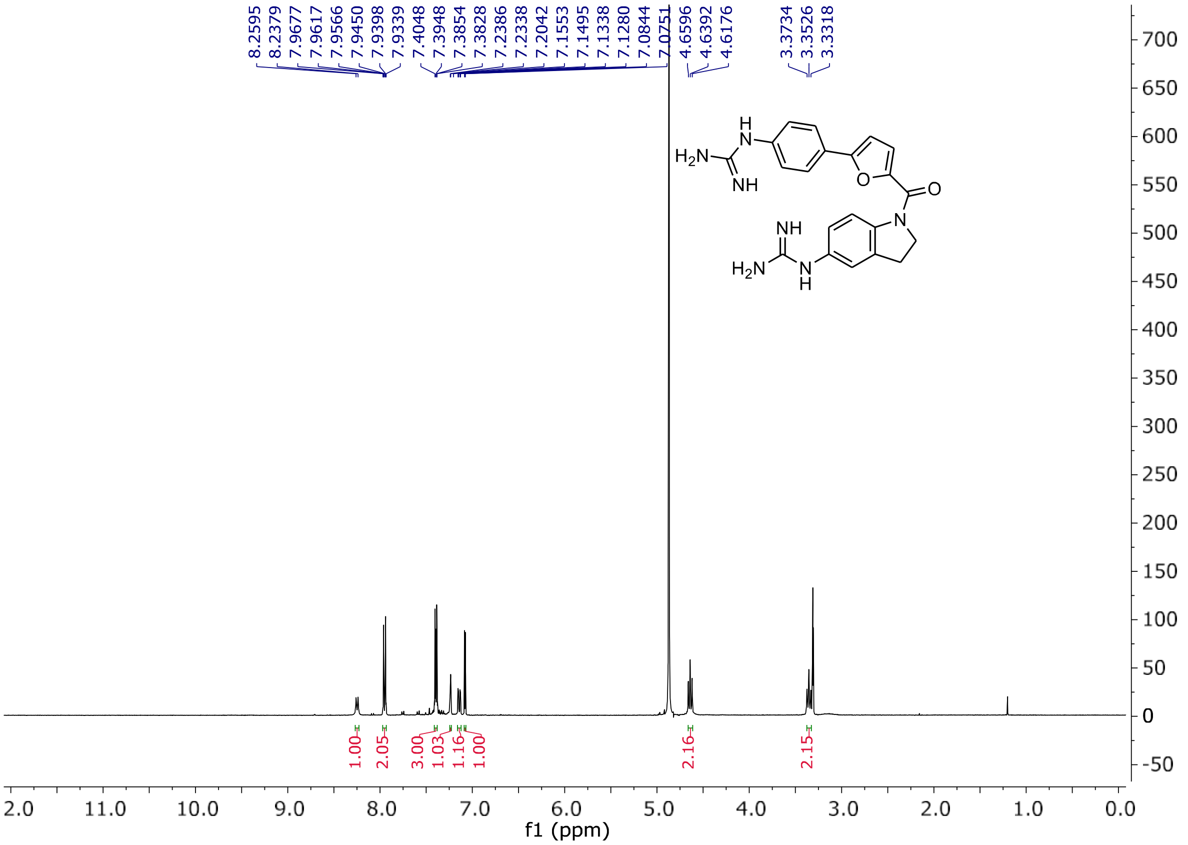

8.2595
8.2379
7.9677
7.9617
7.9566
7.9450
7.9398
7.9339
7.4048
7.3948
7.3854
7.3828
7.2386
7.2338
7.2042
7.1553
7.1495
7.1338
7.1280
7.0844
7.0751
4.6596
4.6392
4.6176
3.3734
3.3526
3.3318
H
N
H2N
NH
O
O
N
NH
H2N
N
H
1.00
2.05
3.00
1.03
1.16
1.00
2.16
2.15
f1 (ppm)

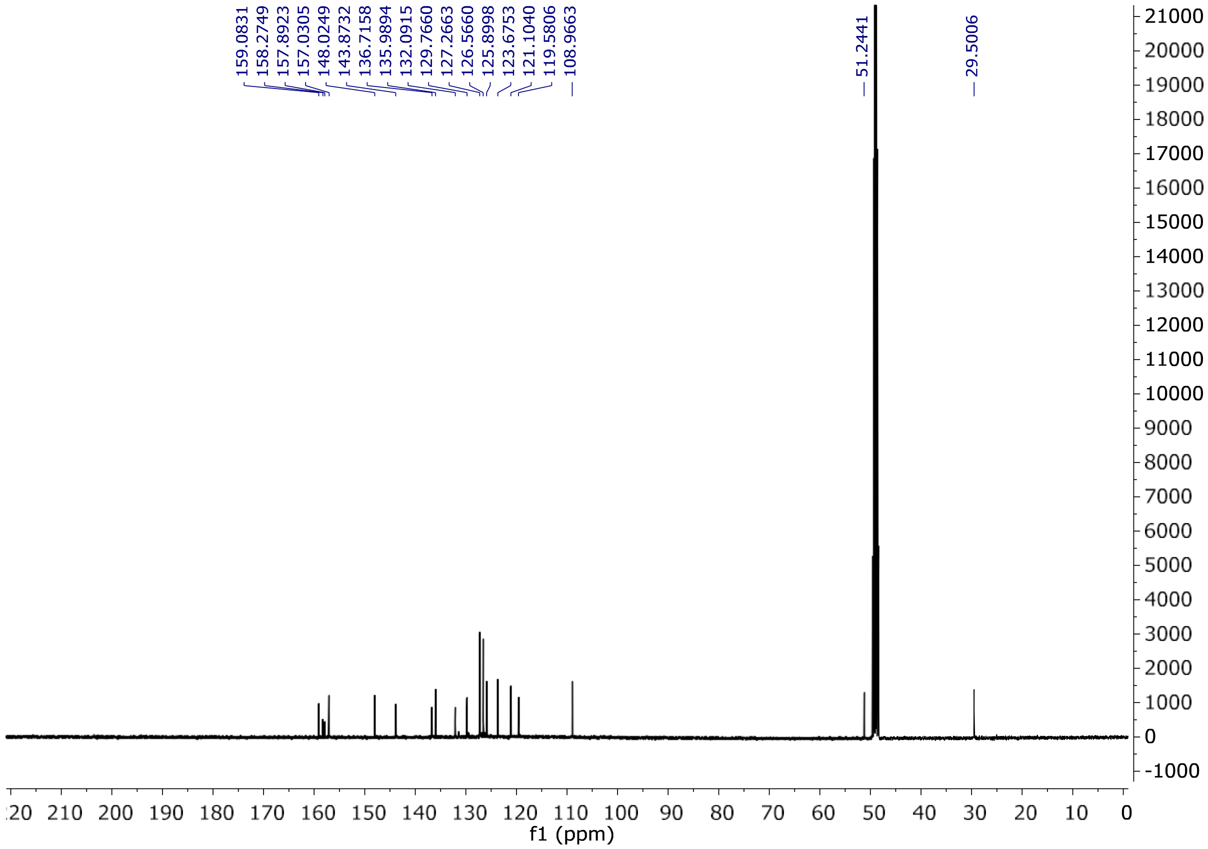

159.0831
158.2749
157.8923
157.0305
148.0249
143.8732
136.7158
135.9894
132.0915
129.7660
127.2663
126.5660
125.8998
123.6753
121.1040
119.5806
108.9663
51.2441
29.5006
f1 (ppm)

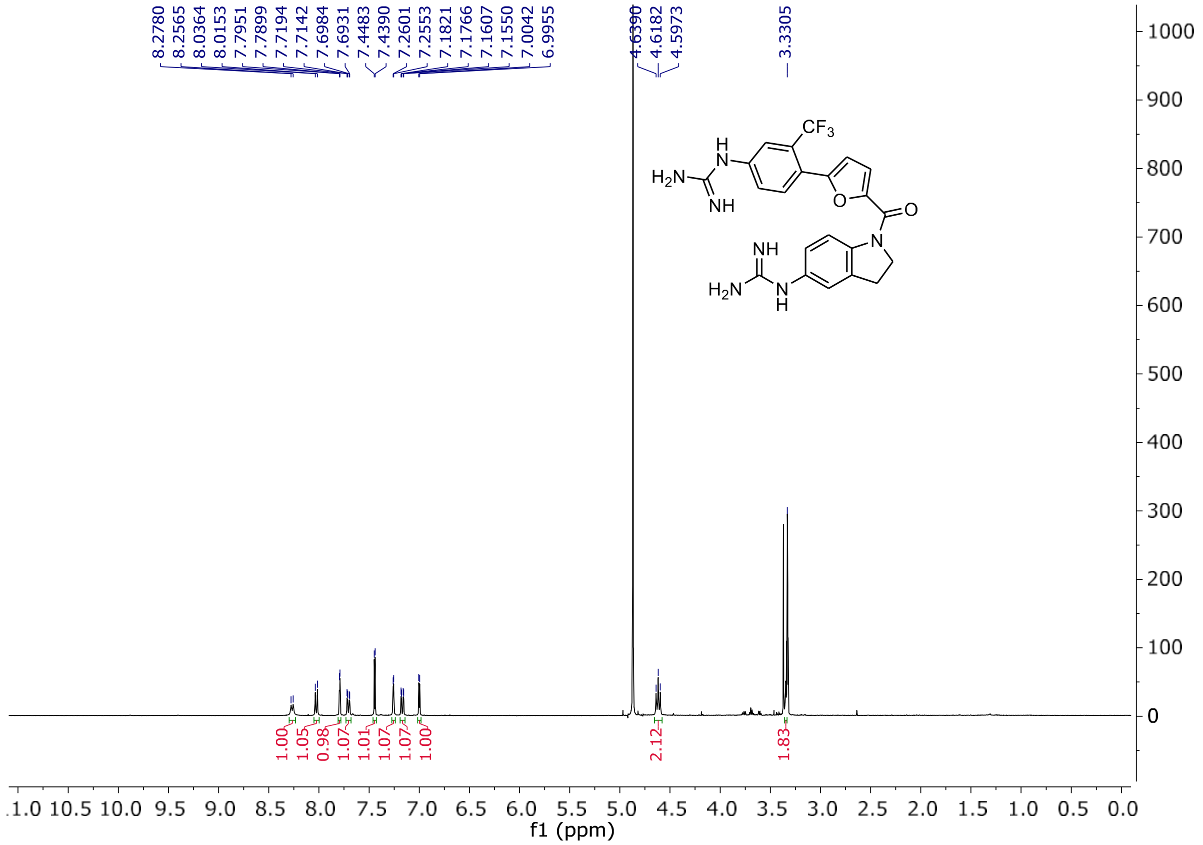
8.2780
8.2565
8.0364
8.0153
7.7951
7.7899
7.7194
7.7142
7.6984
7.6931
7.4483
7.4390
7.2601
7.2553
7.1821
7.1766
7.1607
7.1550
7.0042
6.9955
4.6390
4.6182
4.5973
3.3305
CF3
H
N
H2N
NH
O
O
N
NH
H2N
N
H
1.00
1.05
0.98
1.07
1.01
1.07
1.07
1.00
2.12
1.83
f1 (ppm)

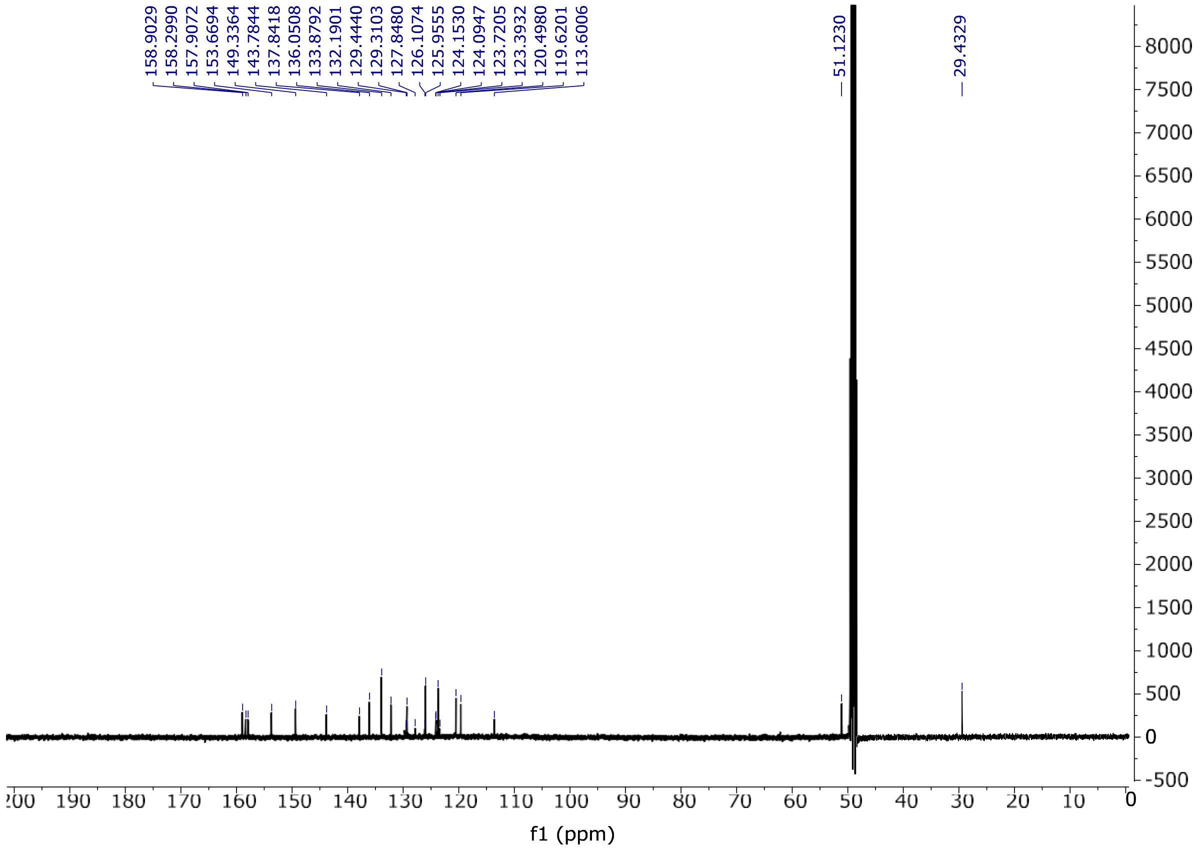
158.9029
158.2990
157.9072
153.6694
149.3364
143.7844
137.8418
136.0508
133.8792
132.1901
129.4440
129.3103
127.8480
126.1074
125.9555
124.1530
124.0947
123.7205
123.3932
120.4980
119.6201
113.6006
51.1230
29.4329
f1 (ppm)

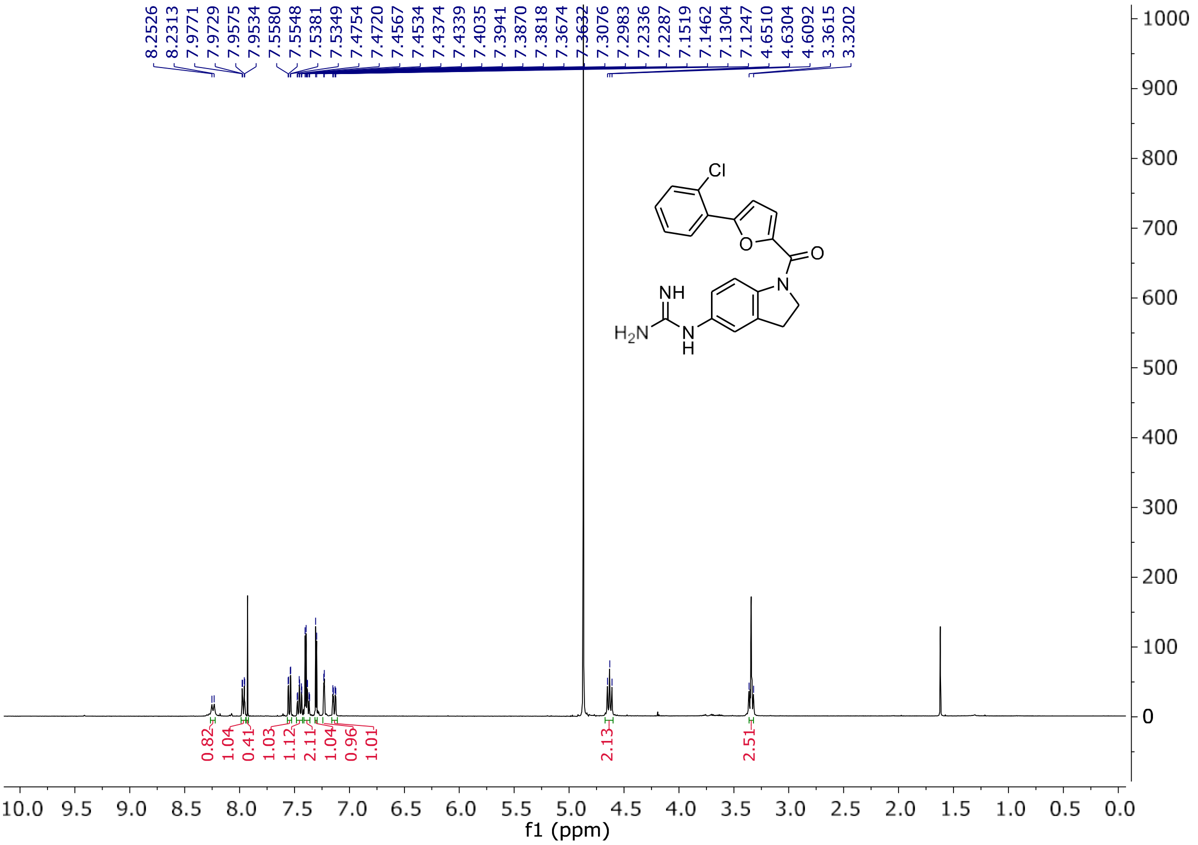
8.2526
8.2313
7.9771
7.9729
7.9575
7.9534
7.5580
7.5548
7.5381
7.5349
7.4754
7.4720
7.4567
7.4534
7.4374
7.4339
7.4035
7.3941
7.3870
7.3818
7.3674
7.3632
7.3076
7.2983
7.2336
7.2287
7.1519
7.1462
7.1304
7.1247
4.6510
4.6304
4.6092
3.3615
3.3202
Cl
O
O
N
NH
H_2N
N
H
0.82
1.04
0.41
1.03
1.12
2.11
1.04
0.96
1.01
2.13
2.51
f1 (ppm)

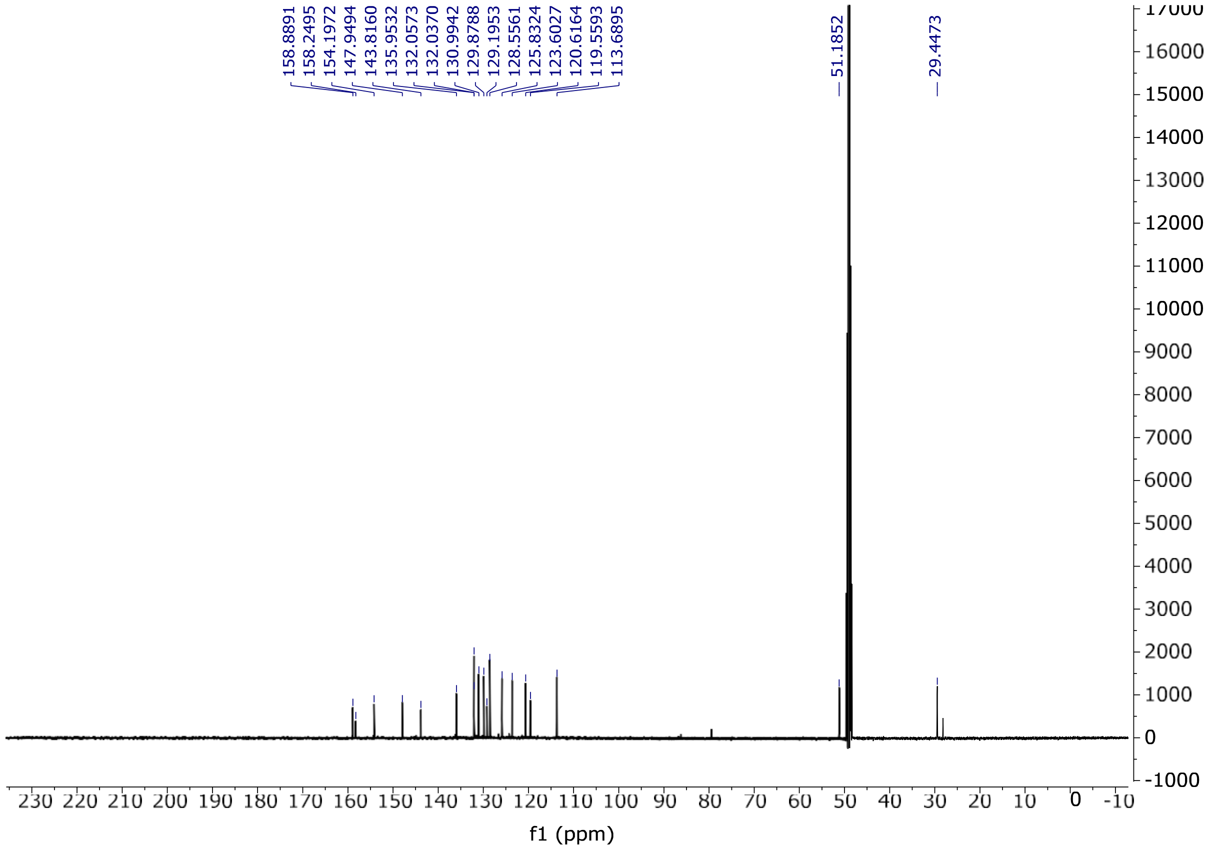
158.8891
158.2495
154.1972
147.9494
143.8160
135.9532
132.0573
132.0370
130.9942
129.8788
129.1953
128.5561
125.8324
123.6027
120.6164
119.5593
113.6895
51.1852
29.4473
f1 (ppm)

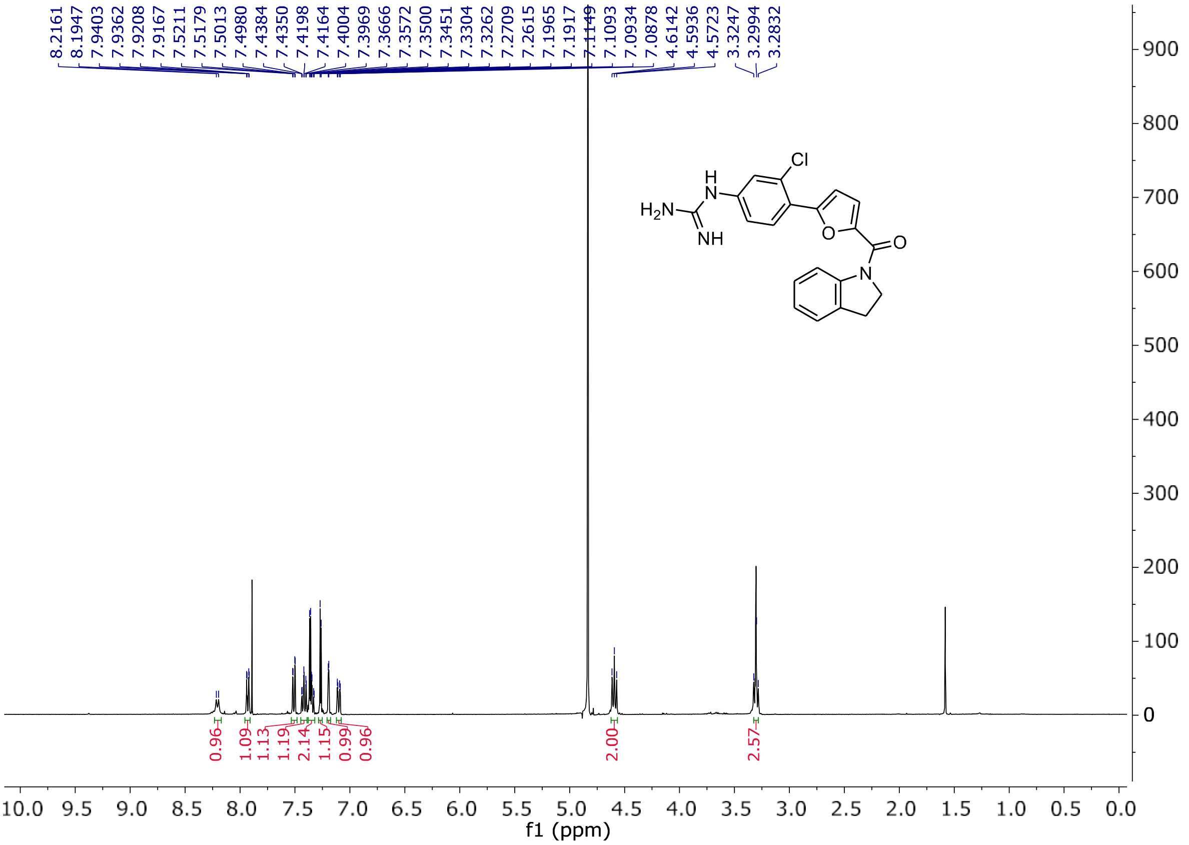
8.2161
8.1947
7.9403
7.9362
7.9208
7.9167
7.5211
7.5179
7.5013
7.4980
7.4384
7.4350
7.4198
7.4164
7.4004
7.3969
7.3666
7.3572
7.3500
7.3451
7.3304
7.3262
7.2709
7.2615
7.1965
7.1917
7.1149
7.1093
7.0934
7.0878
4.6142
4.5936
4.5723
3.3247
3.2994
3.2832
Cl
H
N
H_2N
NH
O
O
N
0.96
1.09
1.13
1.19
2.14
1.15
0.99
0.96
2.00
2.57
f1 (ppm)

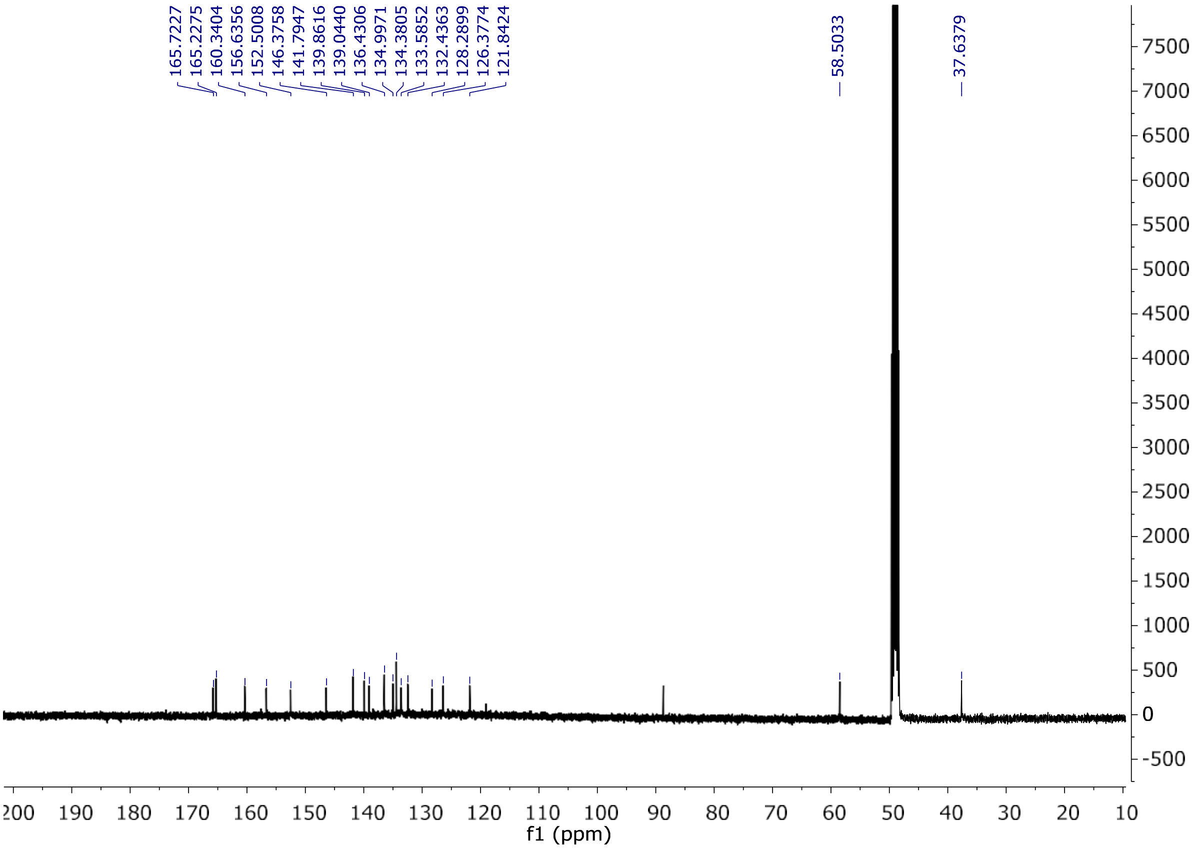
165.7227
165.2275
160.3404
156.6356
152.5008
146.3758
141.7947
139.8616
139.0440
136.4306
134.9971
134.3805
133.5852
132.4363
128.2899
126.3774
121.8424
58.5033
37.6379
f1 (ppm)
